# Supplementary material for: Smoker, ex-smoker or non-smoker? The validity of routinely recorded smoking status in UK primary care: a cross-sectional study
Source: BMJ Open. 2014 Apr 23;4(4):e004958. doi: 10.1136/bmjopen-2014-004958 (PMC4010810; doi:10.1136/bmjopen-2014-004958)
Supplement: Author's manuscript [file bmjopen-2014-004958.draft_revisions.pdf]

Smoker, ex-smoker or non-smoker? The validity of routinely recorded smoking status in UK primary care: a cross-sectional study

Journal:	*BMJ Open*
Manuscript ID:	bmjopen-2014-004958
Article Type:	Research
Date Submitted by the Author:	30-Jan-2014

Complete List of Authors:	Marston, Louise; University College London, Primary Care and Population Health<br>Carpenter, James; London School of Hygiene & Tropical Medicine, Medical Statistics; MRC Clinical Trials Unit,<br>Walters, Kate; University College London, Primary Care and Population Health<br>Morris, Richard; University College London, Primary Care and Population Health<br>Nazareth, Irwin; University College London, Primary Care and Population Health<br>White, Ian; Medical Research Council, Biostatistics Unit<br>Petersen, Irene; University College London, Primary Care and Population Health

**Primary Subject Heading**:	General practice / Family practice
Secondary Subject Heading:	Smoking and tobacco, Research methods
Keywords:	PRIMARY CARE, STATISTICS & RESEARCH METHODS, EPIDEMIOLOGY

SCHOLARONE™
Manuscripts

Title: Smoker, ex-smoker or non-smoker? The validity of routinely recorded smoking status in UK primary care: a cross-sectional study

Authors

Louise Marston[1]

James R Carpenter[2, 3]

Kate R Walters[1]

Richard W Morris[1]

Irwin Nazareth[1,]

Ian R White[4]

Irene Petersen[1]

[1]Department of Primary Care and Population Health, University College London, Rowland Hill Street, London, NW3 2PF, United Kingdom

[2]Department of Medical Statistics, London School of Hygiene and Tropical Medicine, Keppel Street, London, WC1E 7HT, United Kingdom

[3] MRC Clinical Trials Unit, Kingsway, London

[4]MRC Biostatistics Unit, Cambridge Institute of Public Health, University Forvie Site, Robinson Way, Cambridge, CB2 0SR, United Kingdom

Please address correspondence to:

Dr Louise Marston

Department of Primary Care and Population Health

For peer review only

University College London

Rowland Hill Street

London

NW3 2PF

United Kingdom

l.marston@ucl.ac.uk

Telephone: +44 20 7794 0500 (36768)

Fax: +44 20 7794 1224

Words: 3,166

For peer review only

For peer review only - http://bmjopen.bmj.com/site/about/guidelines.xhtml

ABSTRACT

Objectives: To investigate how smoking status is recorded in UK primary care; to evaluate if multiple imputation (MI) of smoking status can give results consistent with health surveys.

Setting: UK primary care and a population survey conducted in the community.

Participants: We identified 354,204 patients aged 16 or over in The Health Improvement Network (THIN) primary care database registered with their general practice 2008-2009 and 15,102 individuals aged 16 or over in the Health Survey for England (HSE).

Outcome measures: Age-standardised and, age-specific proportions of smokers, ex-smokers and non-smokers in THIN and the HSE before and after multiple imputation (MI). Using information on time since quitting in the HSE, we extrapolated when ex-smokers may be considered as non-smokers in primary care.

Results: In THIN, smoking status was recorded for 84% of patients within one year of registration. Of these; 28% were smokers (21% in the HSE). After MI of missing smoking data, the proportion of smokers was 25% (missing at random) and 20% (missing not at random). With increasing age, more were identified as ex-smokers in the HSE than THIN. It appears that those who quit before the age

For peer review only - http://bmjopen.bmj.com/site/about/guidelines.xhtml

of 25-30 years were less likely to be recorded as an ex-smoker in primary care than people who quit later.

Conclusions: Smoking status is relatively well recorded in primary care. Misclassification of ex-smokers as non-smokers is likely to occur in those quitting smoking at an early age and/ or a long time ago. Those with no smoking status information are more likely to be ex or non-smokers versus smokers.

STRENGTHS AND LIMITATIONS OF THIS STUDY

- This study includes data from 'real' life primary care electronic records
- First study to compare the definition of smoking status in primary care versus a population survey
- Study focuses on data recorded in the first year after patient registration and may not be applicable to other times.

KEYWORDS: recording of smoking, primary care databases, Health Survey for England, missing data, multiple imputation

For peer review only

INTRODUCTION

A fifth of the British adult population are smokers [1] and there is still a need for further research into smoking and smoking related diseases including coronary heart disease and stroke, respiratory diseases and cancers. Routinely collected smoking data can be used in clinical practice to identify populations at risk of smoking-related diseases, such as identifying smokers to have spirometry testing to identify those with Chronic Obstructive Pulmonary Disease (COPD), or to be invited for smoking cessation services. It is important to understand the accuracy of the data, and whether cases may be missed in those with no recorded smoking status. Electronic health records, including primary care databases, have proved to be very powerful resources for epidemiological and health research.[2-12] In order to conduct such research, it is important to understand how smoking status is recorded in primary care. There is evidence that the recording of smoking status has improved substantially in UK primary care[13, 14] and most general practices now routinely record smoking status at regular intervals as a part of the Quality Outcome Framework.[15] However, we do not know how the different and non-standardised classifications of ex, non and current smokers in primary care records compare to the standardised recording of smoking status in population surveys such as the Health Survey for England (HSE).

In addition, a proportion of patients still lack a smoking status record in their primary care records. It is unclear how to deal with these patients when

For peer review only

conducting research where smoking status is either the outcome of the research or an explanatory factor for patients' health.[3, 6, 16, 17] Methodological research has demonstrated that including only patients with complete records can substantially bias the results.[18] In recent years, efforts have been made to address missing data in primary care databases[3, 17, 19] using multiple imputation, though reporting on the comparability of the results of multiple imputation with population data has been sparse. Therefore, it is unclear whether multiple imputation accurately replicates data representing the population.[3, 6, 17, 20] Our previous work on missing data in The Health Improvement Network (THIN) primary care database showed that many health indicator measurements (for example, weight and blood pressure) recorded within the first year of patients' registration at a general practice were comparable with large external datasets before and after multiple imputation.[16] However, smoking status was not directly comparable with data from the Health Survey for England (HSE). Although the proportion of smokers was similar between THIN and the HSE *before* multiple imputation of data in THIN, the proportion of smokers was substantially higher *after* multiple imputation in THIN. On the other hand, the proportion of ex-smokers was substantially lower in THIN both *before* and *after* imputation compared to the HSE. This suggests that current smokers may be adequately identified using primary care data and most people with missing data on smoking status are likely to be either ex or non-smokers. This has clinical importance as smoking status (including ex-smoking) may be used to

For peer review only

For peer review only - http://bmjopen.bmj.com/site/about/guidelines.xhtml

identify those at risk of disease, for example chronic obstructive pulmonary disease or cardiovascular disease.

In this study we further investigate recording of smoking status in primary care and explore potential reasons for the discrepancy in the proportion of ex-smokers between primary care records and the HSE. Specifically, we seek to deduce when ex-smokers may not be recorded as such in primary care records based on information about time since quitting in the HSE. Finally, we aim to provide a practical solution for imputation of missing smoking status records in routinely collected clinical data.

METHODS

Study populations

We used data from THIN primary care database.[21] In the United Kingdom (UK) 98% of the population are registered with a National Health Service (NHS) general practitioner to receive routine healthcare.[22] THIN is broadly representative of all general practices in the UK in terms of age and sex of patients, practice size and geographical distribution.[23] The database contains information on socio demographics, symptoms, diagnoses, referrals to secondary care, prescribing, results of tests and health status indicators. The data provider (CSD-MR) obtained overall ethical approval from the South East MREC (MREC/03/01/073) and this study was further approved by a THIN scientific review committee.

For peer review only - http://bmjopen.bmj.com/site/about/guidelines.xhtml

For this study we selected patients aged 16 years or over who registered with a general practice between 1st January 2008 and 31st December 2009 (N=354,204) and we examined records from the first year after the patient registered, hence using data up to the end of 2010. Many people have a "new patient check" soon after registration, where information on demographics, health indicators and disease status is collected.

We compared the distribution of smoking status with that in the HSE from 2008 for those aged 16 years or over (N=15,102). The HSE is a national annual cross sectional interview based survey of approximately 22,000 people.[24] The survey includes questions on socio demographics, general health and information on smoking status. The HSE has nearly complete records of smoking (99.3%) and we therefore used the data from patients with complete smoking information.

Definition of smoking status

In THIN, smoking status was recorded by self-report. In many general practices this would be on the basis of a questionnaire submitted at the time of registration, whereas in other general practices this would be recorded in conjunction with a clinical consultation with the general practitioner or practice nurse. Patients would be classed as current non-smoker, or current smokers. In some instance the non-smokers would be classified as ex-smokers but this was variably defined

For peer review only - http://bmjopen.bmj.com/site/about/guidelines.xhtml

from one practice to another. In the HSE, smoking status was defined on the basis of a series of questions (see Appendix 1) and individuals who had ever smoked (but did not smoke at the time of the interview) would be defined as ex-smokers, regardless of their age at quitting and length of time since they quit. The HSE holds information on when ex-smokers quit so that age at the time they quit can be deduced, whereas this information was not consistently available in THIN.

Statistical analyses

Initially, we examined smoking status (smoker, ex-smoker, non-smoker or missing) in THIN and the HSE, overall, by age group, gender and Index of Multiple Deprivation 2004 (IMD) quintile[25]. Then we used multiple imputation to impute missing smoking status in THIN. Multiple imputation is a statistical method which uses the data available to model the likely distribution of missing data.[18] A number of imputed datasets are produced in each of which plausible values are drawn from the imputation model. The method is designed to correctly reflect the uncertainty surrounding the missing values. With an appropriate imputation model, multiple imputation is an unbiased method of accounting for missing data. It is usually performed under the missing at random (MAR) assumption, but it may also be performed under specific missing not at random (MNAR) assumptions. These methods have been described in greater detail elsewhere.[18, 26-28]

For peer review only

After preliminary analysis,[26] we included the following variables in the multiple imputation models: age in years, gender and IMD quintile,[25] health indicators: smoking status (three categories, non, ex and current smoker), height, weight, systolic and diastolic blood pressures and disease indicators: type II diabetes, coronary heart disease (CHD) and cerebrovascular accident (CVA). Multiple imputation was performed using Chained Equations using the ice command using Stata 11.[29, 30] Continuous variables were imputed using multiple linear regression, smoking status using multinomial regression and IMD quintile using ordered logistic regression. Percentages in each smoking category were obtained using Rubin's Rules.[31] In the first multiple imputation we assumed that smoking data were MAR and hence allowed imputed smoking data of either smokers, non-smokers or ex-smokers (using a MAR assumption; hereafter referred to as MAR MI). In the second multiple imputation we assumed that all smokers had been recorded (so that smoking data were MNAR) and we imputed missing smoking data as either ex-smokers or non-smokers (hereafter referred to as MNAR MI).

Following multiple imputation we carried out age-specific direct standardisation using the HSE as the standard population and the age-specific proportion in each smoking category from THIN. This was done to account for the fact that the mean age in the HSE was 49 years while the mean age in THIN was 38 years in the year after registration.

For peer review only - http://bmjopen.bmj.com/site/about/guidelines.xhtml

We deduced the average time after which an ex-smoker is no longer classified as an ex-smoker in primary care records by combining information from the HSE on when ex-smokers quit and the age-specific distribution of ex-smokers in THIN after imputation of non and ex-smokers. This was done by ranking the individuals in the HSE in accordance to the length of time since they quit by 10 year age groups and then 'reclassifying' individuals who had quit the longest time ago within each age group from ex to non until we reached the same proportion of ex-smokers in the HSE as in THIN.

RESULTS

In total, 354,204 individuals were included from THIN and 15,102 from the HSE. Individuals in THIN were, on average 11 years younger than those in the HSE (38 years versus 49 years, respectively) (Table 1). Smoking status was recorded for 84% in THIN within one year of initial registration. Before multiple imputation of missing data, a greater proportion of people were recorded as smokers in THIN than the HSE (24% versus 21% respectively), and the proportions of ex-smokers and non-smokers differed substantially between THIN and the HSE (Table 1).

For peer review only

For peer review only - http://bmjopen.bmj.com/site/about/guidelines.xhtml

Table 1: Summary statistics for THIN in the first year of registration and the HSE 2008

	THIN		HSE	
**Variable**	**n**	**%**	**n**	**%**
Male	164,085	46	6,760	45
Female	190,119	54	8,342	55
Missing sex		0		0
Non-smoker	165,618	47	7,874	52
Ex-smoker	49,874	14	3,966	26
Current smoker	83,526	24	3,158	21
Missing smoking status	55,186	16	104	1
Age years mean (SD)	38	(17)	49	(19)
Missing age		0		0

Abbreviations: HSE Health Survey for England 2008; THIN The Health Improvement Network.

Our first analyses used missing as a separate category of smoking, so we refer to those with reported smoking status as “known smokers” and “known ex-smokers”. The proportion of known smokers by age group was similar in THIN and the HSE between 30 and 79 years, but this was not the case for the proportions of known ex-smokers and non-smokers (Figure 1). In the HSE, the proportion of ex-smokers increased from 12% within the 20-29 age group to 46% in the 80-89 age group. In THIN, the proportion of known ex-smokers also increased with age although the overall proportion of known ex-smokers was smaller than in the HSE for all age groups after 20-29 years. Conversely, in the HSE, the proportion of non-smokers decreased slightly from 56% in the 20-29 age group to 48% in the 80-89 age group. Within THIN, the proportion of known non-smokers remained constant with increasing age at around 43%. The proportion of missing smoking data in THIN was relatively constant at less than

For peer review only

20% until the 70-79 years age group, but increased substantially thereafter (Figure 1).

(Figure 1 here)

In THIN, the percentage of non-smokers was greater for women (52%) than men (40%) while the percentage of smokers was smaller for women (21%) than men (27%). There were similar trends in the HSE, although the percentage differences between sexes were smaller (smokers: 22% of men versus 20% of women).

The proportions in each smoking status category varied substantially by social deprivation in both THIN and the HSE (Figure 2). In THIN, the percentage of non-smokers decreased from 52% in the least deprived quintile to 40% in the most deprived quintile. The percentage of ex-smokers decreased slightly with increasing deprivation. In contrast, the percentage of smokers increased with increasing deprivation from 16% in the least deprived quintile to 34% in the most deprived quintile (Figure 2). The patterns were similar in the HSE although the proportion of ex-smokers was substantially larger across all levels of deprivation in the HSE compared to THIN.

(Figure 2 here)

For peer review only - http://bmjopen.bmj.com/site/about/guidelines.xhtml

Analyses imputing missing smoking status

After MAR MI of THIN, age-standardised smoking prevalences still differed somewhat between THIN and the HSE. For example, 22% were ex-smokers in THIN compared with 26% in the HSE; 25% were smokers in THIN, compared with 21%in the HSE (Table 2).

After MNAR MI of THIN (that is, regarding missing values as either ex-smokers or non-smokers), the age-standardised prevalence of smoking in THIN was similar to that in the HSE (Table 2). However, the age-specific prevalence of ex-smokers was still greater in HSE than in THIN. Age-specific analysis showed that this difference was greatest at older ages, and indeed reversed at younger ages This suggested that individuals who had quit in the less recent past might be classified as non-smokers in THIN but as ex-smokers in HSE.(Figure 3).

Table 2: Percentages within each smoking status for THIN and the HSE 2008 after various adjustments

Category	THIN			HSE	
	Complete case	After MAR MI [ab]	After MNAR MI [ac]	Observed	Reclassifying ex-smokers [d]
	%	%	%	%	%
Non-smoker	55	53	57	53	57
Ex-smoker	17	22	23	26	22
Smoker	28	25	20	21	21

Abbreviations: HSE Health Survey for England 2008; THIN The Health Improvement Network.

[a] Directly standardised using the HSE age distribution as standard.

[b] Imputed assuming that missing values are smokers, non-smokers or ex-smokers

[c] Imputed assuming that missing values are non-smokers or ex-smokers

[d] Within each age group, reclassifying the optimum number of ex-smokers as non-smokers.

For peer review only - http://bmjopen.bmj.com/site/about/guidelines.xhtml

(Figure 3 here)

The median time since ex-smokers quit in the HSE varied greatly by age group (Table 3), from two years (Interquartile range (IQR): 0, 3) in the under 20s to 40 (IQR: 25, 51) years in those aged 90 or over (Table 3). Equating proportions of ex-smokers in THIN to that in the HSE data suggested the typical time-window after which patients are no longer regarded as ex-smokers in primary care, but instead regarded as non-smokers, varied with age. Thus, typically individuals who registered with a general practice when they were in their forties would no longer be recorded as an ex-smoker if they quit more than 22 years earlier (when they were between 18 and 27 years of age) (Table 3). Individuals registering in their seventies would typically no longer be recorded as ex-smokers if they quit 42 years earlier (when they were between the ages of 28 and 37 years) (Table 3). Yet, most individuals who quit after the age of 30 would still be captured as ex-smokers when they later registered with a new general practice. Using these age-specific extrapolations to reclassify ex-smokers as non-smokers in the HSE according to when they quit, we can see that the age-specific distributions of ex-smokers in THIN and the reclassified HSE are similar (Figure 3).

For peer review only

For peer review only - http://bmjopen.bmj.com/site/about/guidelines.xhtml

Table 3: Age specific centiles of time since quitting smoking in the HSE 2008

Age group	Median time since quitting (years)	Extrapolated number of years since quitting	Extrapolated age when they quit
<20	2	*	*
20-29	3	*	*
30-39	5	14	16 - 25
40-49	10	22	18 - 27
50-59	20	30	20 - 29
60-69	24	35	25 - 34
70-79	30	42	28 - 37
80-89	32	40	40 - 49
90+	40	46	44+

*Not possible to assign an optimal value for reclassification to these age groups
Abbreviations: HSE Health Survey for England 2008

DISCUSSION

The proportion of newly registered patients in THIN between 2008 and 2009 with a record of being a smoker was slightly higher than the HSE in 2008. However, the proportion of individuals recorded as ex-smokers and non-smokers differed substantially between THIN and the HSE. Overall, a larger proportion of individuals were recorded as ex-smokers in the HSE than in THIN and this increased with age. Likewise, the proportion of ex-smokers was substantially larger across all levels of deprivation in the HSE compared to THIN.

Under MAR MI there was a greater percentage of smokers (25%) and a smaller percentage of ex-smokers (22%) in THIN compared with the HSE (smokers 21%, ex-smokers 26%). However, under MNAR MI (assuming all missing data were either ex-smokers or non-smokers) slightly increased the proportion of non-smokers (57%) in THIN compared to the HSE (53%), whereas the proportion of ex-smokers (23%) was slightly lower in THIN. Moreover, the latter imputation

For peer review only - http://bmjopen.bmj.com/site/about/guidelines.xhtml

resulted in a relatively larger percentage of ex-smokers in THIN in those aged under 30 years compared with the HSE. This may be because the imputation model was unable to distinguish between ex and non-smokers in those age groups as both are unlikely to have developed typical later onset diseases which are key predictors of smoking status in the imputation model.

There may be several reasons for the discrepancy in the distribution of the smoking categories between THIN and the HSE. In the HSE, the definition of an ex-smoker was highly sensitive and clearly defined.[24] Thus respondents were categorised as ex-smokers even if they were a trivial smoker, smoked for a short period of time and/ or quit many decades ago. Also, the HSE used computer aided personal interviewing; where questions were read to the respondent in a standardised way from the screen and a detailed sequence of questions were asked to ascertain current smoking status. In primary care, while smoking status is systematically recorded in medical records, there is no detailed protocol for recording smoking status and the ascertainment is thus likely to vary by how the information was obtained. Many practices use self-report questionnaires at registration including smoking status. Smoking status is then updated by health professionals (general practitioners and/ or practice nurses) during consultations where smoking status is often recorded as part of an assessment of current or future disease risk.

For peer review only

For peer review only - http://bmjopen.bmj.com/site/about/guidelines.xhtml

Our examination of the age-standardised data suggests that typically an ex-smoker in primary care settings is recorded as a non-smoker when they quit at a young age or had not smoked for a substantial time period. This could be because the patient may not volunteer previous smoking in either initial self-report questionnaire or on questioning by clinicians when it was minor, long ago or they consider it not relevant to their current or future health. It is possible that patients are more reluctant to volunteer ex-smoking habits when data are being held on their medical record and is not anonymous. However, comparing the proportion of individuals with a smoking record in THIN with that of the HSE we found a similar distribution suggesting that most smokers were identified in the first year of their registration in primary care. Similar findings have been observed in the literature by calendar year.[32] While some studies suggest underreporting of smoking among pregnant women in primary care[33] we found no evidence this was a general pattern. With the introduction of the Quality and Outcomes Framework in 2004, there has also been increased incentive to identify smokers in relation to specific disease outcomes.[34, 35] Indeed we found in our previous study that those with respiratory and cardiac conditions were more likely to have any smoking status recorded within the first year of registration.[13] Smoking status was validated in the HSE in 2007 by the use of saliva cotinine samples and was found to be accurate[36].

The method of age standardisation then deducing the average time since quitting and reclassifying them to non-smokers in the HSE is relatively crude and

For peer review only - http://bmjopen.bmj.com/site/about/guidelines.xhtml

assumes that everyone who becomes an ex-smoker does so at the same time in their lives as others in their age group. However, it may be indicative of reporting of smoking status at the GP practice, given the results shown in this study.

An alternative method of dealing with unobserved smoking data is to dichotomise smoking status into current smokers and non-current smokers with missing data assumed to be non-current smokers. However, it should be noted that this solution may be to the detriment of some epidemiological studies where ex-smokers who quit recently are at greater risk of disease than non-smokers. For example, the 50 year follow up of male British doctors shows that ex-smokers had elevated age standardised mortality rates for many diseases.[37, 38]

Our findings suggest that *in contrast* to health surveys, patients who quit smoking at a young age (before 25-30) are likely to be recorded by their general practice as a non-smoker instead of an ex-smoker. This has implications for researchers using these data sources. To our knowledge this is the first study which seeks to deduce and quantify typical time between when a smoker quit and when they are no longer perceived as an ex-smoker in primary care. Clinicians, policy-makers and researchers who wish to use smoking status in primary care records to identify populations at risk of smoking-related diseases can be reassured by our findings that using data from new registrations, most current smokers will be identified and misclassification of ex-smokers is more likely to have occurred in those who have quit smoking at an early age and/ or a long time ago.

For peer review only - http://bmjopen.bmj.com/site/about/guidelines.xhtml
For peer review only

Figure Legends

Figure 1: Smoking status percentages in THIN and the HSE 2008 by age group

Figure 1 footnotes:
Solid line is the Health Survey for England 2008, dashed line is The Health Improvement Network (THIN)

Figure 2: Smoking status percentages in THIN and the HSE 2008 by deprivation quintile

Figure 2 footnotes:
*IMD 1 is the least deprived and IMD 5 is the most deprived
Darker bars represent the HSE 2008, lighter bars represent THIN
Abbreviations: HSE Health Survey for England 2008, IMD Index of multiple deprivation, THIN The Health Improvement Network

Figure 3: Age group specific percentages of ex-smokers in THIN (after MNAR imputation) and the HSE 2008 (before and after reclassifying ex-smokers in the HSE who quit before the age specified in Table 3 column 3 to be non-smokers)

Figure 3 footnotes:
Abbreviations: THIN The Health Improvement Network, HSE Health Survey for England 2008

For peer review only

Conflict of interest and funding

The authors have no conflicts of interest to declare. This study is funded by a UK Medical Research Council grant [G0900701]. The funder had no influence over the study design, results or decision to publish this work. JRC was funded by a UK Economic and Social Research Council research fellowship grant [RES-063-27-0257]. IRW was funded by a United Kingdom Medical Research Council grant [U105260558].

Author contributions

LM extracted and analysed the data and wrote the first draft of the paper with help from IP and JRC. KRW and IN provided clinical input and IRW and RWM provided additional statistical input. All authors commented on the paper and helped write subsequent drafts.

Data sharing statement

No data are available

I Dr Louise Marston the Corresponding Author of this article contained within the original manuscript which includes any diagrams & photographs, other illustrative material, video, film or any other material howsoever submitted by the Contributor(s) at any time and related to the Contribution ("the Contribution") have the right to grant on behalf of all authors and do grant on behalf of all authors, a licence to the BMJ Publishing Group Ltd and its licensees, to permit this Contribution (if accepted) to be published in BMJ Open and any other BMJ Group products and to exploit all subsidiary rights, as set out in the licence at: (http://group.bmj.com/products/journals/instructions-for-authors/BMJOpen_licence.pdf)

For peer review only - http://bmjopen.bmj.com/site/about/guidelines.xhtml

References

1 Office for National Statistics Opinions and Lifestyle Survey, Smoking Habits Amongst Adults, 2012; 2013 (http://www.ons.gov.uk/ons/dcp171776_328041.pdf) (accessed January 2014)

2 Davies AR, Smeeth L, Grundy EMD. Contribution of changes in incidence and mortality to trends in the prevalence of coronary heart disease in the UK: 1996-2005. *Eur Heart J* 2007;2142-2147.

3 Delaney JAC, Daskalopoulou SS, Brophy JM et al Lifestyle variables and the risk of myocardial infarction in the general practice research database (electronic article). *BMC Cardiovasc Disord* 2007;38.

4 Douglas IJ, Smeeth L. Exposure to antipsychotics and risk of stroke: self controlled case series study (electronic article). *Br Med J* 2008;

5 Gelfand JM, Neimann AL, Shin DB et al Risk of myocardial infarction in patients with psoriasis. *JAMA* 2006;1735-1741.

6 Hippisley-Cox J, Coupland C, Vinogradova Y et al Derivation and validation of QRISK, a new cardiovascular disease risk score for the United Kingdom: prospective open cohort study. *Br Med J* 2007;136-141.

For peer review only - http://bmjopen.bmj.com/site/about/guidelines.xhtml

7 Osborn DPJ, Levy G, Nazareth I et al Relative risk of cardiovascular and cancer mortality in people with severe mental illness from the United Kingdom's General Practice Research Database. *Arch Gen Psychiatry* 2007;242-249.

8 Smeeth L, Thomas SL, Hall AJ et al Risk of myocardial infarction and stroke after acute infection or vaccination. *N Engl J Med* 2004;2611-2618.

9 Walters K, Rait G, Petersen I et al Panic disorder and risk of new onset coronary heart disease, acute myocardial infarction, and cardiac mortality: cohort study using the general practice research database. *Eur Heart J* 2008;2981-2988.

10 Horsfall LJ, Rait G, Walters K et al Serum Bilirubin and Risk of Respiratory Disease and Death *JAMA* 2011;691-697

11 Kiri VA, Fabbri LM, Davis KJ et al Inhaled corticosteroids and risk of lung cancer among COPD patients who quit smoking *Respir Med* 2009;85-90

12 Horsfall LJ, Nazareth I, Petersen I. Cardiovascular Events as a Function of Serum Bilirubin Levels in a Large Statin-Treated Cohort. *Circulation* 2012;2556-2564

For peer review only - http://bmjopen.bmj.com/site/about/guidelines.xhtml

13 Szatkowski L, Lewis S, McNeill A et al Is smoking status routinely recorded when patients register with a new GP? *Fam Pract* 2010;673–675

14 Dhalwani NN, Tata LJ, Coleman T, Fleming KM, Szatkowski L Completeness of Maternal Smoking Status Recording during Pregnancy in United Kingdom Primary Care Data. *PLoS One* 2013;e72218

15 Coleman T, Lewis S, Hubbard R et al Impact of contractual financial incentives on the ascertainment and management of smoking in primary care. *Addiction* 2007;102:803e8.

16 Marston L, Carpenter JR, Walters KR et al Issues in multiple imputation of missing data for large general practice clinical databases *Pharmacoepidemiol Drug Saf* 2010;618–626

17 Weiner MG, Barnhart K, Xie D et al Hormone therapy and coronary heart disease in young women. *Menopause* 2008;86-93.

18 Sterne JAC, White IR, Carlin JB et al. Multiple imputation for missing data in epidemiological and clinical research: potential and pitfalls. *Br Med J* 2009;b2393

For peer review only - http://bmjopen.bmj.com/site/about/guidelines.xhtml

19 Hippisley-Cox J, Coupland C, Vinogradova Y et al Predicting cardiovascular risk in England and Wales: prospective derivation and validation of QRISK2. *Br Med J* 2008;1475-1482

20 Collins GS, Altman DG An independent and external validation of QRISK2 cardiovascular disease risk score: a prospective open cohort study *Br Med J* 2010;c2442

21 The Health Improvement Network The Health Improvement Network. London: The Health Improvement Network; 2014 (http://csdmruk.cegedim.com/) (Accessed January 2014).

22 Lis Y, Mann RD The VAMP Research multi-purpose database in the U.K. *J Clin Epidemiol* 1995;431-443.

23 Blak BT, Thompson M, Dattani H, Bourke A Generalisability of The Health Improvement Network (THIN) database: demographics, chronic disease prevalence and mortality rates. Inform Prim Care 2011;251-255.

24 National Centre for Social Research and University College London. Department of Epidemiology and Public Health, *Health Survey for England, 2008* [computer file]. *2nd Edition.* Colchester, Essex: UK Data Archive [distributor], October 2010. SN: 6397

For peer review only

25 Noble M, Wright G, Dibben C et al Indices of Deprivation 2004. Report to the Office of the Deputy Prime Minister. London: Neighbourhood Renewal Unit; 2004 (http://webarchive.nationalarchives.gov.uk/20120919132719/http://www.communities.gov.uk/documents/communities/pdf/131209.pdf) (Accessed January 2014)

26 Spratt M, Carpenter J, Sterne JAC et al Strategies for Multiple Imputation in Longitudinal Studies *Am. J. Epidemiol.* 2010;478-487

27 White IR, Royston P, Wood A Multiple imputation using chained equations: issues and guidance for practice (tutorial). *Stat Med* 2011;377-399

28 Graham JW Missing data analysis: Making it work in the real world *Annu Rev Psychol* 2009;549-576

29 Stata Corporation. *Stata Statistical Software: Release 11*. College Station, TX: Stata Corporation; 2009

30 Royston P Multiple imputation of missing values: Update of ice. *Stata Journal* 2005;527-536.

31 Rubin DB *Multiple imputation for non-response in surveys*. New York: John Wiley and Sons; 1987

For peer review only

32 Szatkowski L, Lewis S, McNeill A et al Can data from primary care medical records be used to monitor national smoking prevalence? *J Epidemiol Community Health* 2011. doi:10.1136/jech.2010.120154

33 Shipton D, Tappin DM, Vadiveloo T et al Reliability of self reported smoking status by pregnant women for estimating smoking prevalence: a retrospective, cross sectional study *Br Med J* 2009;b4347.

34 The British Medical Association and NHS Employers Quality and Outcomes Framework guidance for GMS contract 2011/12; 2011 (http://www.nhsemployers.org/Aboutus/Publications/Documents/QOF_guidance_GMS_contract_2011_12.pdf) (Accessed January 2014)

35 Campbell S, Reeves D, Kontopantelis E et al Quality of Primary Care in England with the Introduction of Pay for Performance *N Engl J Med* 2007;181-190

36 Wardle H, Mindell J Adult cigarette smoking. In Craig R, Shelton N (Ed.), Health Survey for England 2007. Volume 1. Healthy lifestyles: Knowledge, attitudes and behaviour. 2008;149-176 NHS Information Centre.

For peer review only - http://bmjopen.bmj.com/site/about/guidelines.xhtml

37 Doll R, Peto R, Boreham J et al Mortality in relation to smoking: 50 years' observations on male British doctors *Br Med J* 2004, doi:10.1136/bmj.38142.554479.AE

38 Kenfield SA, Wei EK, Rosner BA et al Burden of smoking on cause-specific mortality: application to the Nurses' Health Study *Tob Control* 2010;248-254

For peer review only

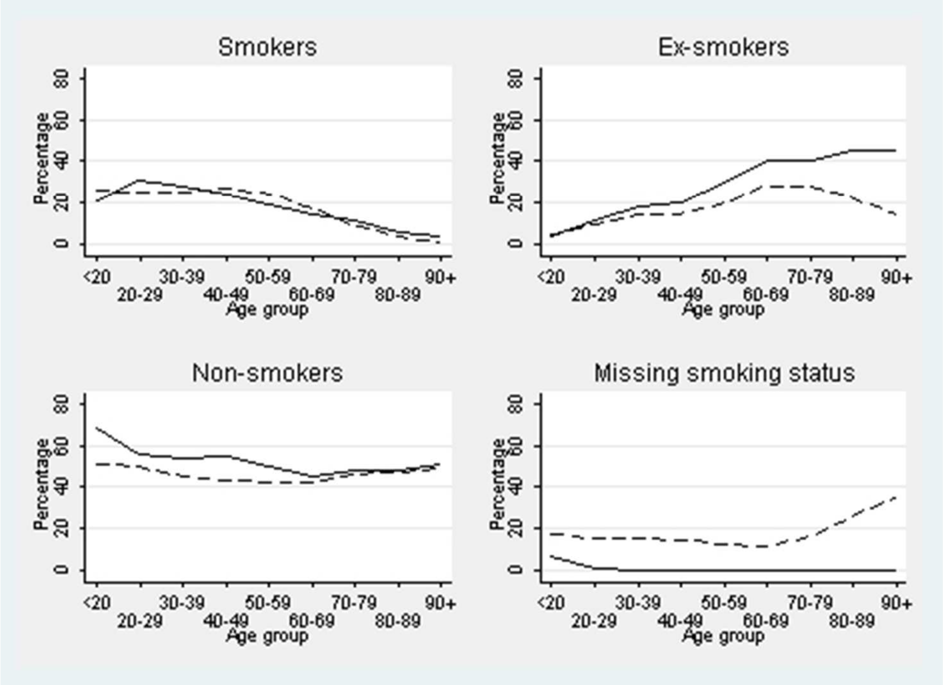

Figure 1: Smoking status percentages in THIN and the HSE 2008 by age group

Solid line is the Health Survey for England 2008, dashed line is The Health Improvement Network (THIN)

166x120mm (72 x 72 DPI)

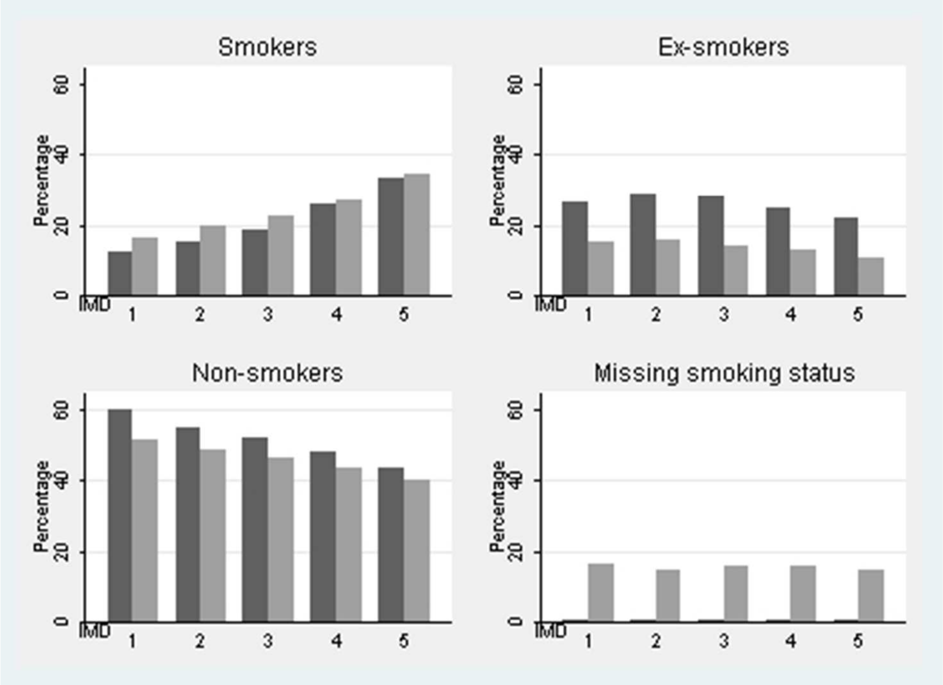

Figure 2: Smoking status percentages in THIN and the HSE 2008 by deprivation quintile

*IMD 1 is the least deprived and IMD 5 is the most deprived
Darker bars represent the HSE 2008, lighter bars represent THIN
Abbreviations: HSE Health Survey for England 2008, IMD Index of multiple deprivation, THIN The Health Improvement Network

166x120mm (72 x 72 DPI)

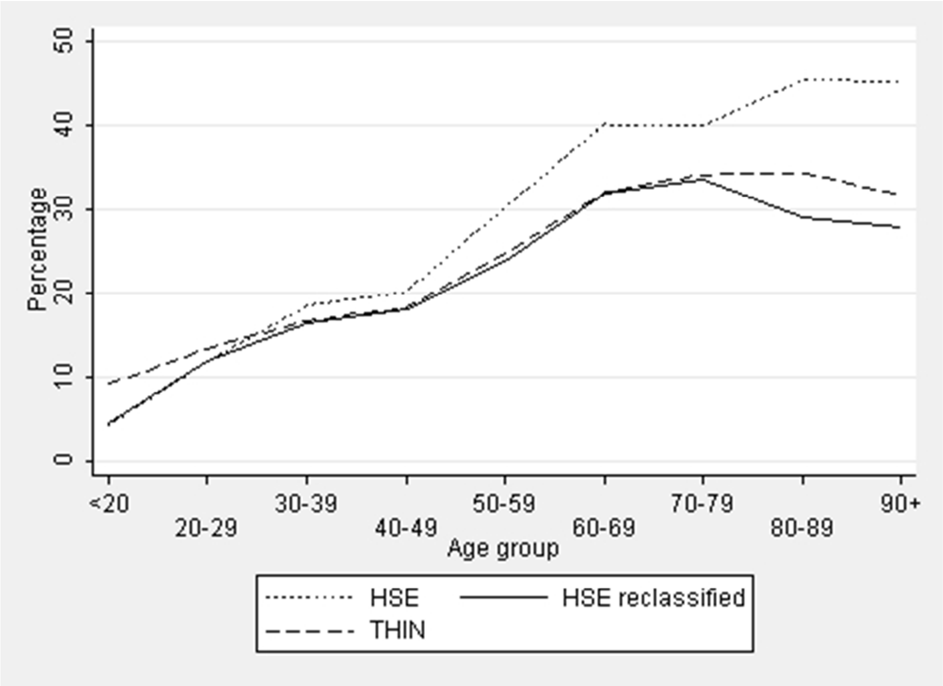

Figure 3: Age group specific percentages of ex-smokers in THIN (after MNAR imputation) and the HSE 2008 (before and after reclassifying ex-smokers in the HSE who quit before the age specified in Table 3 column 3 to be non-smokers)

Abbreviations: THIN The Health Improvement Network, HSE Health Survey for England 2008

166x120mm (72 x 72 DPI)

STROBE 2007 (v4) Statement—Checklist of items that should be included in reports of *cross-sectional studies*

Section/Topic	Item #	Recommendation	Reported on page #
**Title and abstract**	1	(*a*) Indicate the study's design with a commonly used term in the title or the abstract	1
		(*b*) Provide in the abstract an informative and balanced summary of what was done and what was found	3-4
**Introduction**			
Background/rationale	2	Explain the scientific background and rationale for the investigation being reported	5-7
Objectives	3	State specific objectives, including any prespecified hypotheses	7
**Methods**			
Study design	4	Present key elements of study design early in the paper	7-8
Setting	5	Describe the setting, locations, and relevant dates, including periods of recruitment, exposure, follow-up, and data collection	7-8
Participants	6	(*a*) Give the eligibility criteria, and the sources and methods of selection of participants	8
Variables	7	Clearly define all outcomes, exposures, predictors, potential confounders, and effect modifiers. Give diagnostic criteria, if applicable	8-10
Data sources/ measurement	8*	For each variable of interest, give sources of data and details of methods of assessment (measurement). Describe comparability of assessment methods if there is more than one group	8-10
Bias	9	Describe any efforts to address potential sources of bias	*8*
Study size	10	Explain how the study size was arrived at	NA
Quantitative variables	11	Explain how quantitative variables were handled in the analyses. If applicable, describe which groupings were chosen and why	9-10
Statistical methods	12	(*a*) Describe all statistical methods, including those used to control for confounding	9-11
		(*b*) Describe any methods used to examine subgroups and interactions	9-11
		(*c*) Explain how missing data were addressed	9-11
		(*d*) If applicable, describe analytical methods taking account of sampling strategy	NA
		(*e*) Describe any sensitivity analyses	NA
**Results**			

For peer review only

Participants	13*	(a) Report numbers of individuals at each stage of study—eg numbers potentially eligible, examined for eligibility, confirmed eligible, included in the study, completing follow-up, and analysed	11
		(b) Give reasons for non-participation at each stage	NA
		(c) Consider use of a flow diagram	NA
Descriptive data	14*	(a) Give characteristics of study participants (eg demographic, clinical, social) and information on exposures and potential confounders	11-12
		(b) Indicate number of participants with missing data for each variable of interest	12
Outcome data	15*	Report numbers of outcome events or summary measures	12
Main results	16	(*a*) Give unadjusted estimates and, if applicable, confounder-adjusted estimates and their precision (eg, 95% confidence interval). Make clear which confounders were adjusted for and why they were included	12-16
		(*b*) Report category boundaries when continuous variables were categorized	NA
		(*c*) If relevant, consider translating estimates of relative risk into absolute risk for a meaningful time period	NA
Other analyses	17	Report other analyses done—eg analyses of subgroups and interactions, and sensitivity analyses	NA
**Discussion**			
Key results	18	Summarise key results with reference to study objectives	16
Limitations	19	Discuss limitations of the study, taking into account sources of potential bias or imprecision. Discuss both direction and magnitude of any potential bias	17-19
Interpretation	20	Give a cautious overall interpretation of results considering objectives, limitations, multiplicity of analyses, results from similar studies, and other relevant evidence	17-18
Generalisability	21	Discuss the generalisability (external validity) of the study results	19
**Other information**			
Funding	22	Give the source of funding and the role of the funders for the present study and, if applicable, for the original study on which the present article is based	21

*Give information separately for cases and controls in case-control studies and, if applicable, for exposed and unexposed groups in cohort and cross-sectional studies.

Note: An Explanation and Elaboration article discusses each checklist item and gives methodological background and published examples of transparent reporting. The STROBE checklist is best used in conjunction with this article (freely available on the Web sites of PLoS Medicine at http://www.plosmedicine.org/, Annals of Internal Medicine at http://www.annals.org/, and Epidemiology at http://www.epidem.com/). Information on the STROBE Initiative is available at www.strobe-statement.org.

For peer review only

Smoker, ex-smoker or non-smoker? The validity of routinely recorded smoking status in UK primary care: a cross-sectional study

Journal:	*BMJ Open*
Manuscript ID:	bmjopen-2014-004958.R1
Article Type:	Research
Date Submitted by the Author:	20-Mar-2014

Complete List of Authors:	Marston, Louise; University College London, Primary Care and Population Health<br>Carpenter, James; London School of Hygiene & Tropical Medicine, Medical Statistics; MRC Clinical Trials Unit,<br>Walters, Kate; University College London, Primary Care and Population Health<br>Morris, Richard; University College London, Primary Care and Population Health<br>Nazareth, Irwin; University College London, Primary Care and Population Health<br>White, Ian; Medical Research Council, Biostatistics Unit<br>Petersen, Irene; University College London, Primary Care and Population Health

**Primary Subject Heading**:	General practice / Family practice
Secondary Subject Heading:	Smoking and tobacco, Research methods
Keywords:	PRIMARY CARE, STATISTICS & RESEARCH METHODS, EPIDEMIOLOGY

SCHOLARONE™ Manuscripts

For peer review only

Title: Smoker, ex-smoker or non-smoker? The validity of routinely recorded smoking status in UK primary care: a cross-sectional study

Authors

Louise Marston[1]

James R Carpenter[2, 3]

Kate R Walters[1]

Richard W Morris[1]

Irwin Nazareth[1,]

Ian R White[4]

Irene Petersen[1]

[1]Department of Primary Care and Population Health, University College London, Rowland Hill Street, London, NW3 2PF, United Kingdom

[2]Department of Medical Statistics, London School of Hygiene and Tropical Medicine, Keppel Street, London, WC1E 7HT, United Kingdom

[3] MRC Clinical Trials Unit, Kingsway, London

[4]MRC Biostatistics Unit, Cambridge Institute of Public Health, University Forvie Site, Robinson Way, Cambridge, CB2 0SR, United Kingdom

Please address correspondence to:

Dr Louise Marston

Department of Primary Care and Population Health

For peer review only

University College London

Rowland Hill Street

London

NW3 2PF

United Kingdom

l.marston@ucl.ac.uk

Telephone: +44 20 7794 0500 (36768)

Fax: +44 20 7794 1224

Words: 3,376

For peer review only

ABSTRACT

Objectives: To investigate how smoking status is recorded in UK primary care; to evaluate whether appropriate multiple imputation (MI) of smoking status yields results consistent with health surveys.

Setting: UK primary care and a population survey conducted in the community.

Participants: We identified 354,204 patients aged 16 or over in The Health Improvement Network (THIN) primary care database registered with their general practice 2008-2009 and 15,102 individuals aged 16 or over in the Health Survey for England (HSE).

Outcome measures: Age-standardised and age-specific proportions of smokers, ex-smokers and non-smokers in THIN and the HSE before and after multiple imputation (MI). Using information on time since quitting in the HSE, we estimated when ex-smokers are typically recorded as non-smokers in primary care records.

Results: In THIN, smoking status was recorded for 84% of patients within one year of registration. Of these; 28% were smokers (21% in the HSE). After MI of missing smoking data, the proportion of smokers was 25% (missing at random) and 20% (missing not at random). With increasing age, more were identified as ex-smokers in the HSE than THIN. It appears that those who quit before age 30

For peer review only - http://bmjopen.bmj.com/site/about/guidelines.xhtml

were less likely to be recorded as an ex-smoker in primary care than people who quit later.

Conclusions: Smoking status was relatively well recorded in primary care. Misclassification of ex-smokers as non-smokers is likely to occur in those quitting smoking at an early age and/ or a long time ago. Those with no smoking status information are more likely to be ex or non-smokers than smokers.

STRENGTHS AND LIMITATIONS OF THIS STUDY

- This study includes data from 'real' life primary care electronic records
- First study to compare the definition of smoking status in primary care with a population survey
- Study focuses on data recorded in the first year after patient registration and may not be applicable to other times.

KEYWORDS: recording of smoking, primary care databases, Health Survey for England, missing data, multiple imputation

For peer review only

INTRODUCTION

A fifth of the British adult population are smokers [1] and there is still a need for further research into smoking and smoking related diseases including coronary heart disease and stroke, respiratory diseases and cancers. Routinely collected smoking data can be used in clinical practice to identify populations at risk of smoking-related diseases, such as identifying smokers to undergo spirometry testing for early diagnosis of Chronic Obstructive Pulmonary Disease (COPD), or to be invited for smoking cessation services. It is important to understand the accuracy of the data, and whether cases may be missed in those with no recorded smoking status. Electronic health records, including primary care databases, have proved to be very powerful resources for epidemiological and health research.[2-12], allowing research that would be difficult using primary research methods; for example, studying the elderly and people with severe mental illness.[4, 7, 9, 11] Additionally they include millions of patients giving power to study rare conditions. Nevertheless, as they are collected for clinical reasons, they raise a number of issues when used for research; not least of these is missing data.

In order to conduct such research, it is important to understand how smoking status is recorded in primary care and how missing data may be addressed. There is evidence that the recording of smoking status has improved substantially in UK primary care[13, 14] and estimates of *current smoking* are similar to large population surveys.[15, 16] Most general practices now routinely

record smoking status at regular intervals as a part of the Quality Outcome Framework.[17] However, we do not know how the different and non-standardised classifications of *ex and, non smokers* in primary care records compared to the standardised recording of smoking status in population surveys such as the Health Survey for England (HSE).

As noted already, a proportion of patients still lack a smoking status record in their primary care records. It is unclear how to deal with these patients when conducting research where smoking status is either the outcome of the research or an explanatory factor for patients' health.[3, 6, 18, 19] Methodological research has demonstrated that including only patients with complete records can substantially bias the results, especially when the reason for missing data is associated with patient outcomes.[20, 21] In recent years, efforts have been made to address missing data in primary care databases[3, 19, 22] using multiple imputation, though reporting on the comparability of the results of multiple imputation with population data has been sparse. Therefore, it is unclear whether multiple imputation accurately replicates data representing the population.[3, 6, 19, 23] Our previous work on missing data in The Health Improvement Network (THIN) primary care database showed that many health indicator measurements (for example, weight and blood pressure) recorded within the first year of patients' registration at a general practice were comparable with large external datasets before and after multiple imputation.[18] However, smoking status was not directly comparable with data from the Health Survey for

For peer review only - http://bmjopen.bmj.com/site/about/guidelines.xhtml

England (HSE). Although the proportion of smokers was similar between THIN and the HSE *before* multiple imputation of data in THIN, the proportion of smokers was substantially higher *after* multiple imputation in THIN. On the other hand, the proportion of ex-smokers was substantially lower in THIN both *before* and *after* imputation compared to the HSE. This suggests that current smokers may be adequately identified using primary care data and most people with missing data on smoking status are likely to be either ex or non-smokers. This has clinical importance as smoking status (including ex-smoking) may be used to identify those at risk of disease, for example chronic obstructive pulmonary disease or cardiovascular disease.

In this study we further investigate recording of smoking status in primary care and explore potential reasons for the discrepancy in the proportion of ex-smokers between primary care records and the HSE. Specifically, we seek to deduce when ex-smokers may not be recorded as such in primary care records based on information about time since quitting in the HSE. Finally, we aim to provide a practical solution for imputation of missing smoking status records in routinely collected clinical data.

METHODS

Study populations

We used data from THIN primary care database, from practices in England that had passed data quality checks, to ensure they were using their computer

For peer review only - http://bmjopen.bmj.com/site/about/guidelines.xhtml

system to record all patient consultations.[24-26] In the United Kingdom (UK) 98% of the population are registered with a National Health Service (NHS) general practitioner to receive routine healthcare.[27] THIN is broadly representative of all general practices in the UK in terms of age and sex of patients, practice size and geographical distribution.[28] The database contains information on socio demographics, symptoms, diagnoses, referrals to secondary care, prescribing, results of tests and health status indicators. The data provider (CSD-MR) obtained overall ethical approval from the South East MREC (MREC/03/01/073) and this study was further approved by a THIN scientific review committee.

For this study we selected patients aged 16 years or over who registered with a general practice between 1st January 2008 and 31st December 2009 (N=354,204) and were registered for at least a year. We examined records from the first year after the patient registered, hence using data up to the end of 2010. Many people have a “new patient check” soon after registration, where information on demographics, health indicators and disease status is collected.

We compared the distribution of smoking status with that in the HSE from 2008 for those aged 16 years or over (N=15,102). The HSE is a national annual cross sectional interview based survey of approximately 22,000 people.[29] The survey includes questions on socio demographics, general health and information on smoking status. The HSE has nearly complete records of

For peer review only - http://bmjopen.bmj.com/site/about/guidelines.xhtml

smoking (99.3%) and we therefore used the data from patients with complete smoking information.

Definition of smoking status

In THIN, smoking status was recorded by self-report. In many general practices this would be on the basis of a questionnaire submitted at the time of registration, whereas in other general practices this would be recorded in conjunction with a clinical consultation with the general practitioner or practice nurse. GPs and nurses may be more interested in the separation between current non-smokers and smokers, thus the non-smoking categories may include some people who are never smokers as well as some who are ex-smokers in primary care records. . In THIN we extracted smoking status data either using Read codes[30] which were classified into non-smoker, ex-smoker and smoker with clinical input, or we used the categorisation (non-smoker, ex-smoker or current smoker) provided in the Additional Health Data. In the HSE, smoking status was defined on the basis of a series of questions (see Appendix 1) and individuals who had ever smoked (but did not smoke at the time of the interview) would be defined as ex-smokers, regardless of their age at quitting and length of time since they quit. The HSE holds information on when ex-smokers quit so that age at the time they quit can be deduced, whereas this information was not consistently available in THIN.

Statistical analyses

For peer review only

For peer review only - http://bmjopen.bmj.com/site/about/guidelines.xhtml

Initially, we examined smoking status (smoker, ex-smoker, non-smoker or missing) in THIN and the HSE, overall, by age group, gender and Index of Multiple Deprivation 2004 (IMD) quintile[31]. Then we used multiple imputation to impute missing data in THIN. Multiple imputation via full conditional specification was performed using Stata's "ice" command. [32, 33] Multiple imputation is a statistical method which uses the data available to model the likely distribution of missing data.[20] A number of imputed datasets are produced in each of which plausible values are drawn from the imputation model. The method is designed to correctly reflect the uncertainty surrounding the missing values. With an appropriate imputation model, multiple imputation is an unbiased method of accounting for missing data. It is usually performed under the missing at random (MAR) assumption, but it may also be performed under specific missing not at random (MNAR) assumptions. These methods have been described in greater detail elsewhere.[20, 34-36]

After preliminary analysis,[34] we included the following variables in the multiple imputation models: age in years, gender and IMD quintile,[31] health indicators: smoking status (three categories, non, ex and current smoker), height, weight, systolic and diastolic blood pressures and disease indicators: type II diabetes, coronary heart disease (CHD) and cerebrovascular accident (CVA). There were missing values for smoking status, blood pressure, weight, height and IMD quintile. . Within the full conditional specification imputation algorithm, continuous variables were imputed using multiple linear regression, smoking

For peer review only - http://bmjopen.bmj.com/site/about/guidelines.xhtml

status using multinomial regression and IMD quintile using ordered logistic regression. Percentages in each smoking category were obtained using Rubin's Rules.[37] In the first multiple imputation we assumed that smoking data were MAR and hence allowed imputed smoking data of either smokers, non-smokers or ex-smokers (using a MAR assumption; hereafter referred to as MAR MI). In the second multiple imputation we assumed that all smokers had been recorded (so that smoking data were MNAR) and we imputed missing smoking data as either ex-smokers or non-smokers (hereafter referred to as MNAR MI).

Following multiple imputation we carried out age-specific direct standardisation using the HSE as the standard population and the age-specific proportion in each smoking category from THIN. This was done to account for the fact that the mean age in the HSE was 49 years while the mean age in THIN was 38 years in the year after registration.

We deduced the average time after which an ex-smoker is no longer classified as an ex-smoker in primary care records by combining information from the HSE on when ex-smokers quit and the age-specific distribution of ex-smokers in THIN after imputation of non and ex-smokers. This was done by ranking the individuals in the HSE in accordance to the length of time since they quit by 10 year age groups and then 'reclassifying' individuals who had quit the longest time ago within each age group from ex to non until we reached the same proportion of ex-smokers in the HSE as in THIN. By doing this, we were able to estimate

For peer review only - http://bmjopen.bmj.com/site/about/guidelines.xhtml

the average time that elapses from quitting smoking after which true ex-smokers are recorded as non-smokers in primary care records.

RESULTS

In total, 354,204 individuals were included from 366 general practices in THIN and 15,102 individuals from the HSE. Individuals in THIN were, on average 11 years younger than those in the HSE (38 years versus 49 years, respectively) (Table 1). Smoking status was recorded for 84% in THIN within one year of initial registration. Before multiple imputation of missing data, a greater proportion of people were recorded as smokers in THIN than the HSE (24% versus 21% respectively), and the proportions of ex-smokers and non-smokers differed substantially between THIN and the HSE (Table 1).

For peer review only

Table 1: Summary statistics for THIN in the first year of registration and the HSE 2008

	THIN		HSE	
**Variable**	**n**	**%**	**n**	**%**
Male	164,085	46	6,760	45
Female	190,119	54	8,342	55
Missing sex		0		0
Non-smoker	165,618	47	7,874	52
Ex-smoker	49,874	14	3,966	26
Current smoker	83,526	24	3,158	21
Missing smoking status	55,186	16	104	1
Age years mean (SD)	38	(17)	49	(19)
Missing age		0		0
Least deprived	69,104	20	3,321	22
Quintile 2	71,771	20	3,039	20
Quintile 3	66,422	19	3,010	20
Quintile 4	71,789	20	2,928	19
Most deprived	52,120	15	2,804	19
Missing IMD	22,998	6	0	0

Abbreviations: HSE Health Survey for England 2008; THIN The Health Improvement Network.

Our first analyses used missing as a separate category of smoking, so we refer to those with reported smoking status as “known smokers” and “known ex-smokers”. The proportion of known smokers by age group was similar in THIN and the HSE between 30 and 79 years, but this was not the case for the proportions of known ex-smokers and non-smokers (Figure 1). In the HSE, the proportion of ex-smokers increased from 12% within the 20-29 age group to 46% in the 80-89 age group. In THIN, the proportion of known ex-smokers also increased with age although the overall proportion of known ex-smokers was smaller than in the HSE for all age groups after 20-29 years. Conversely, in the HSE, the proportion of non-smokers decreased slightly from 56% in the 20-29

For peer review only

age group to 48% in the 80-89 age group. Within THIN, the proportion of known non-smokers remained constant with increasing age at around 43%. The proportion of missing smoking data in THIN was relatively constant at less than 20% until the 70-79 years age group, but increased substantially thereafter (Figure 1).

(Figure 1 here)

In THIN, the percentage of non-smokers was greater for women (52%) than men (40%) while the percentage of known smokers was smaller for women (21%) than men (27%). There were similar trends in the HSE, although the percentage differences between sexes were smaller (smokers: 22% of men versus 20% of women).

The proportions in each smoking status category varied substantially by social deprivation in both THIN and the HSE (Figure 2). In THIN, the percentage of non-smokers decreased from 52% in the least deprived quintile to 40% in the most deprived quintile. The percentage of known ex-smokers decreased slightly with increasing deprivation. In contrast, the percentage of known smokers increased with increasing deprivation from 16% in the least deprived quintile to 34% in the most deprived quintile (Figure 2). The patterns were similar in the HSE although the proportion of ex-smokers was substantially larger across all levels of deprivation in the HSE compared to THIN.

For peer review only

(Figure 2 here)

Analyses imputing missing smoking status

After MAR MI of THIN, age-standardised smoking prevalences still differed somewhat between THIN and the HSE. For example, 22% were ex-smokers in THIN compared with 26% in the HSE; 25% were smokers in THIN, compared with 21% in the HSE (Table 2).

After MNAR MI of THIN (that is, specifying that missing values are either ex-smokers or non-smokers), the age-standardised prevalence of smoking in THIN was similar to that in the HSE (Table 2). However, the age-specific prevalence of ex-smokers was still greater in the HSE than in THIN. Age-specific analysis showed that this difference was greatest at older ages, and indeed reversed at younger ages. This suggested that individuals who had quit in the less recent past might be classified as non-smokers in THIN but as ex-smokers in HSE.(Figure 3).

For peer review only - http://bmjopen.bmj.com/site/about/guidelines.xhtml

Table 2: Percentages within each smoking status for THIN and the HSE 2008 after various adjustments

Category	THIN			HSE	
	Complete records	After MAR MI. [ab]	After MNAR MI [ac]	Observed	Reclassifying ex-smokers [d]
	%	%	%	%	%
Non-smoker	55	53	57	53	57
Ex-smoker	17	22	23	26	22
Smoker	28	25	20	21	21

Abbreviations: HSE Health Survey for England 2008; THIN The Health Improvement Network.

[a] Directly standardised using the HSE age distribution as standard.

[b] Imputed assuming that missing values are smokers, non-smokers or ex-smokers

[c] Imputed assuming that missing values are non-smokers or ex-smokers

[d] Within each age group, reclassifying the optimum number of ex-smokers as non-smokers based on the distributions shown after MNAR MI.

(Figure 3 here)

The median time since ex-smokers quit in the HSE varied greatly by age group (Table 3), from two years (Interquartile range (IQR): 0, 3) in the under 20s to 40 (IQR: 25, 51) years in those aged 90 or over (Table 3). Equating proportions of ex-smokers in THIN to that in the HSE data suggested the typical time-window after which patients are no longer regarded as ex-smokers in primary care, but instead regarded as non-smokers, varied with age. Thus, typically individuals who registered with a general practice when they were in their forties would no longer be recorded as an ex-smoker if they quit more than 22 years earlier (when they were between 18 and 27 years of age) (Table 3). Individuals registering in their seventies would typically no longer be recorded as ex-smokers if they quit 42 years earlier (when they were between the ages of 28 and 37 years) (Table 3). Yet, most individuals who quit after the age of 30 would still be captured as

For peer review only

ex-smokers when they later registered with a new general practice. Using these age-specific extrapolations to reclassify ex-smokers as non-smokers in the HSE according to when they quit, we can see that the age-specific distributions of ex-smokers in THIN and the reclassified HSE are similar (Figure 3).

Table 3: Age specific centiles of time since quitting smoking in the HSE 2008

Age group	Median time since quitting (years)	Extrapolated number of years since quitting	Extrapolated age when they quit
<20	2	*	*
20-29	3	*	*
30-39	5	14	16 - 25
40-49	10	22	18 - 27
50-59	20	30	20 - 29
60-69	24	35	25 - 34
70-79	30	42	28 - 37
80-89	32	40	40 - 49
90+	40	46	44+

*Not possible to assign an optimal value for reclassification to these age groups
Abbreviations: HSE Health Survey for England 2008

DISCUSSION

The proportion of newly registered patients in THIN between 2008 and 2009 with a record of being a smoker was slightly higher than the HSE in 2008. However, the proportion of individuals recorded as ex-smokers and non-smokers differed substantially between THIN and the HSE. Overall, a larger proportion of individuals were recorded as ex-smokers in the HSE than in THIN and this increased with age. Likewise, the proportion of ex-smokers was substantially larger across all levels of deprivation in the HSE compared to THIN.

For peer review only - http://bmjopen.bmj.com/site/about/guidelines.xhtml

Under MAR MI there was a greater percentage of smokers (25%) and a smaller percentage of ex-smokers (22%) in THIN compared with the HSE (smokers 21%, ex-smokers 26%). However, MNAR MI (assuming all missing data were either ex-smokers or non-smokers) slightly increased the proportion of non-smokers (57%) in THIN compared to the HSE (53%), whereas the proportion of ex-smokers (23%) was slightly lower in THIN. Moreover, the latter imputation resulted in a relatively larger percentage of ex-smokers in THIN in those aged under 30 years compared with the HSE. This may be because the imputation model was unable to distinguish between ex and non-smokers in those age groups as both are unlikely to have developed typical later onset diseases which are key predictors of smoking status in the imputation model.

There may be several reasons for the discrepancy in the distribution of the smoking categories between THIN and the HSE. In the HSE, the definition of an ex-smoker was highly sensitive and clearly defined.[29] Thus respondents were categorised as ex-smokers even if they were a trivial smoker, smoked for a short period of time and/ or quit many decades ago. Also, the HSE used computer aided personal interviewing; where questions were read to the respondent in a standardised way from the screen and a detailed sequence of questions were asked to ascertain current smoking status. In primary care, while smoking status is systematically recorded in medical records, there is no detailed protocol for recording smoking status and the ascertainment is thus likely to vary by how the information was obtained. Many practices use self-report questionnaires at

For peer review only - http://bmjopen.bmj.com/site/about/guidelines.xhtml

registration including smoking status. Smoking status is then updated by health professionals (general practitioners and/ or practice nurses) during consultations where smoking status is often recorded as part of an assessment of current or future disease risk.

Our examination of the age-standardised data suggests that typically an ex-smoker in primary care settings is recorded as a non-smoker when they quit at a young age or had not smoked for a substantial time period. This could be because the patient may not volunteer previous smoking in either initial self-report questionnaire or on questioning by clinicians when it was minor, long ago or they consider it not relevant to their current or future health. It is possible that patients are more reluctant to volunteer ex-smoking habits when data are being held on their medical record and is not anonymous. However, comparing the proportion of individuals with a smoking record in THIN with that of the HSE we found a similar distribution suggesting that most smokers were identified in the first year of their registration in primary care. Similar findings have been observed in the literature by calendar year.[18] With the introduction of the Quality and Outcomes Framework in 2004, there has also been increased incentive to identify smokers in relation to specific disease outcomes.[38, 39] Indeed we found in our previous study that those with respiratory and cardiac conditions were more likely to have any smoking status recorded within the first year of registration.[13] Smoking status was validated in the HSE in 2007 by the use of saliva cotinine samples and was found to be accurate[40].

For peer review only

The method of age standardisation then deducing the average time since quitting and reclassifying them to non-smokers in the HSE is relatively crude and assumes that everyone who becomes an ex-smoker does so at the same time in their lives as others in their age group. However, it is likely to be indicative of reporting of smoking status at the GP practice, given the results shown in this study.

An alternative method of dealing with unobserved smoking data is to dichotomise smoking status into current smokers and non-current smokers with missing data assumed to be non-current smokers. However, it should be noted that this solution may be to the detriment of some epidemiological studies where ex-smokers who quit recently are at greater risk of disease than non-smokers. For example, the 50 year follow up of male British doctors shows that ex-smokers had elevated age standardised mortality rates for many diseases.[41, 42]

Our findings suggest that *in contrast* to health surveys, patients who quit smoking at a young age (before 25-30) are likely to be recorded by their general practice as a non-smoker instead of an ex-smoker. This has implications for researchers using these data sources. To our knowledge this is the first study which seeks to deduce and quantify typical time between when a smoker quit and when they are no longer perceived as an ex-smoker in primary care. Clinicians, policy-makers and researchers who wish to use smoking status in primary care records to

For peer review only - http://bmjopen.bmj.com/site/about/guidelines.xhtml

identify populations at risk of smoking-related diseases can be reassured by our findings that using data from new registrations, most current smokers will be identified and misclassification of ex-smokers is more likely to have occurred in those who have quit smoking at an early age and/ or a long time ago.

For peer review only

For peer review only - http://bmjopen.bmj.com/site/about/guidelines.xhtml

Conflict of interest and funding

The authors have no conflicts of interest to declare. This study is funded by a UK Medical Research Council grant [G0900701]. The funder had no influence over the study design, results or decision to publish this work. JRC was funded by a UK Economic and Social Research Council research fellowship grant [RES-063-27-0257]. IRW was funded by a United Kingdom Medical Research Council grant [U105260558].

Author contributions

LM extracted and analysed the data and wrote the first draft of the paper with help from IP and JRC. KRW and IN provided clinical input and IRW and RWM provided additional statistical input. All authors commented on the paper and helped write subsequent drafts.

Data sharing statement

No additional data available.

I Dr Louise Marston the Corresponding Author of this article contained within the original manuscript which includes any diagrams & photographs, other illustrative material, video, film or any other material howsoever submitted by the Contributor(s) at any time and related to the Contribution ("the Contribution") have the right to grant on behalf of all authors and do grant on behalf of all authors, a licence to the BMJ Publishing Group Ltd and its licensees, to permit this Contribution (if accepted) to be published in BMJ Open and any other BMJ Group products and to exploit all subsidiary rights, as set out in the licence at: (http://group.bmj.com/products/journals/instructions-for-authors/BMJOpen_licence.pdf)

For peer review only - http://bmjopen.bmj.com/site/about/guidelines.xhtml

References

1 Office for National Statistics Opinions and Lifestyle Survey, Smoking Habits Amongst Adults, 2012; 2013 (http://www.ons.gov.uk/ons/dcp171776_328041.pdf) (accessed January 2014)

2 Davies AR, Smeeth L, Grundy EMD. Contribution of changes in incidence and mortality to trends in the prevalence of coronary heart disease in the UK: 1996-2005. *Eur Heart J* 2007;2142-2147.

3 Delaney JAC, Daskalopoulou SS, Brophy JM et al Lifestyle variables and the risk of myocardial infarction in the general practice research database (electronic article). *BMC Cardiovasc Disord* 2007;38.

4 Douglas IJ, Smeeth L. Exposure to antipsychotics and risk of stroke: self controlled case series study (electronic article). *Br Med J* 2008;

5 Gelfand JM, Neimann AL, Shin DB et al Risk of myocardial infarction in patients with psoriasis. *JAMA* 2006;1735-1741.

6 Hippisley-Cox J, Coupland C, Vinogradova Y et al Derivation and validation of QRISK, a new cardiovascular disease risk score for the United Kingdom: prospective open cohort study. *Br Med J* 2007;136-141.

For peer review only

7 Osborn DPJ, Levy G, Nazareth I et al Relative risk of cardiovascular and cancer mortality in people with severe mental illness from the United Kingdom's General Practice Research Database. *Arch Gen Psychiatry* 2007;242-249.

8 Smeeth L, Thomas SL, Hall AJ et al Risk of myocardial infarction and stroke after acute infection or vaccination. *N Engl J Med* 2004;2611-2618.

9 Walters K, Rait G, Petersen I et al Panic disorder and risk of new onset coronary heart disease, acute myocardial infarction, and cardiac mortality: cohort study using the general practice research database. *Eur Heart J* 2008;2981-2988.

10 Horsfall LJ, Rait G, Walters K et al Serum Bilirubin and Risk of Respiratory Disease and Death *JAMA* 2011;691-697

11 Kiri VA, Fabbri LM, Davis KJ et al Inhaled corticosteroids and risk of lung cancer among COPD patients who quit smoking *Respir Med* 2009;85-90

12 Horsfall LJ, Nazareth I, Petersen I. Cardiovascular Events as a Function of Serum Bilirubin Levels in a Large Statin-Treated Cohort. *Circulation* 2012;2556-2564

For peer review only

13 Szatkowski L, Lewis S, McNeill A et al Is smoking status routinely recorded when patients register with a new GP? *Fam Pract* 2010;673–675

14 Dhalwani NN, Tata LJ, Coleman T, et al. Completeness of Maternal Smoking Status Recording during Pregnancy in United Kingdom Primary Care Data. *PLoS One* 2013;e72218

15 Szatkowski L, Lewis S, McNeill A et al Can data from primary care medical records be used to monitor national smoking prevalence? *J Epidemiol Community Health* 2012;791-795.

16 Langley TE, Szatkowski LC, Wythe S, et al Can primary care data be used to monitor regional smoking prevalence? An analysis of The Health Improvement Network primary care data *BMC Public Health* 2011, 11:773

17 Coleman T, Lewis S, Hubbard R et al Impact of contractual financial incentives on the ascertainment and management of smoking in primary care. *Addiction* 2007;102:803e8.

18 Marston L, Carpenter JR, Walters KR et al Issues in multiple imputation of missing data for large general practice clinical databases *Pharmacoepidemiol Drug Saf* 2010;618–626

For peer review only

For peer review only - http://bmjopen.bmj.com/site/about/guidelines.xhtml

19 Weiner MG, Barnhart K, Xie D et al Hormone therapy and coronary heart disease in young women. *Menopause* 2008;86-93.

20 Sterne JAC, White IR, Carlin JB et al. Multiple imputation for missing data in epidemiological and clinical research: potential and pitfalls. *Br Med J* 2009;b2393

21 Carpenter J, Kenward M Multiple imputation and its application 2013 Wiley

22 Hippisley-Cox J, Coupland C, Vinogradova Y et al Predicting cardiovascular risk in England and Wales: prospective derivation and validation of QRISK2. *Br Med J* 2008;1475-1482

23 Collins GS, Altman DG An independent and external validation of QRISK2 cardiovascular disease risk score: a prospective open cohort study *Br Med J* 2010;c2442

24 The Health Improvement Network The Health Improvement Network. London: The Health Improvement Network; 2014 (http://csdmruk.cegedim.com/) (Accessed January 2014).

25 Horsfall L, Walters K, Petersen I.Identifying periods of acceptable computer usage in primary care research databases. *Pharmacoepidemiol Drug Saf* 2013; 64-69

For peer review only

26 Maguire A, Blak BT, Thompson M. The importance of defining periods of complete mortality reporting for research using automated data from primary care. *Pharmacoepidem Drug Safe* 2009; 76-83.

27 Lis Y, Mann RD The VAMP Research multi-purpose database in the U.K. *J Clin Epidemiol* 1995;431-443.

28 Blak BT, Thompson M, Dattani H, et al Generalisability of The Health Improvement Network (THIN) database: demographics, chronic disease prevalence and mortality rates. *Inform Prim Care* 2011;251-255.

29 National Centre for Social Research and University College London. Department of Epidemiology and Public Health, *Health Survey for England, 2008* [computer file]. *2nd Edition.* Colchester, Essex: UK Data Archive [distributor], October 2010. SN: 6397

30 Booth N What are the Read Codes? *Health Libr Rev* 1994;177-82

31 Noble M, Wright G, Dibben C et al Indices of Deprivation 2004. Report to the Office of the Deputy Prime Minister. London: Neighbourhood Renewal Unit; 2004 (http://webarchive.nationalarchives.gov.uk/20120919132719/http://www.communities.gov.uk/documents/communities/pdf/131209.pdf) (Accessed January 2014)

For peer review only

32 Stata Corporation. *Stata Statistical Software: Release 11*. College Station, TX: Stata Corporation; 2009

33 Royston P Multiple imputation of missing values: Update of ice. *Stata Journal* 2005;527-536.

34 Spratt M, Carpenter J, Sterne JAC et al Strategies for Multiple Imputation in Longitudinal Studies *Am. J. Epidemiol.* 2010;478-487

35 White IR, Royston P, Wood A Multiple imputation using chained equations: issues and guidance for practice (tutorial). *Stat Med* 2011;377-399

36 Graham JW Missing data analysis: Making it work in the real world *Annu Rev Psychol* 2009;549-576

37 Rubin DB *Multiple imputation for non-response in surveys*. New York: John Wiley and Sons; 1987

38 The British Medical Association and NHS Employers Quality and Outcomes Framework guidance for GMS contract 2011/12; 2011 (http://www.nhsemployers.org/Aboutus/Publications/Documents/QOF_guidance_GMS_contract_2011_12.pdf) (Accessed January 2014)

For peer review only

39 Campbell S, Reeves D, Kontopantelis E et al Quality of Primary Care in England with the Introduction of Pay for Performance *N Engl J Med* 2007;181-190

40 Wardle H, Mindell J Adult cigarette smoking. In Craig R, Shelton N (Ed.), Health Survey for England 2007. Volume 1. Healthy lifestyles: Knowledge, attitudes and behaviour. 2008;149-176 NHS Information Centre.

41 Doll R, Peto R, Boreham J et al Mortality in relation to smoking: 50 years' observations on male British doctors *Br Med J* 2004, doi:10.1136/bmj.38142.554479.AE

42 Kenfield SA, Wei EK, Rosner BA et al Burden of smoking on cause-specific mortality: application to the Nurses' Health Study *Tob Control* 2010;248-254

For peer review only

Figure Legends

Figure 1: Smoking status percentages in THIN and the HSE 2008 by age group

Figure 1 footnotes:
Solid line is the Health Survey for England 2008, dashed line is The Health Improvement Network (THIN)

Figure 2: Smoking status percentages in THIN and the HSE 2008 by deprivation quintile

Figure 2 footnotes:
*IMD 1 is the least deprived and IMD 5 is the most deprived
Darker bars represent the HSE 2008, lighter bars represent THIN
Abbreviations: HSE Health Survey for England 2008, IMD Index of multiple deprivation, THIN The Health Improvement Network

Figure 3: Age group specific percentages of ex-smokers in THIN (after MNAR imputation) and the HSE 2008 (before and after reclassifying ex-smokers in the HSE who quit before the age specified in Table 3 column 3 to be non-smokers)

Figure 3 footnotes:
Abbreviations: THIN The Health Improvement Network, HSE Health Survey for England 2008

For peer review only

For peer review only - http://bmjopen.bmj.com/site/about/guidelines.xhtml

Title: Smoker, ex-smoker or non-smoker? The validity of routinely recorded smoking status in UK primary care: a cross-sectional study

Authors

Louise Marston[1]

James R Carpenter[2, 3]

Kate R Walters[1]

Richard W Morris[1]

Irwin Nazareth[1,]

Ian R White[4]

Irene Petersen[1]

[1]Department of Primary Care and Population Health, University College London, Rowland Hill Street, London, NW3 2PF, United Kingdom

[2]Department of Medical Statistics, London School of Hygiene and Tropical Medicine, Keppel Street, London, WC1E 7HT, United Kingdom

[3] MRC Clinical Trials Unit, Kingsway, London

[4]MRC Biostatistics Unit, Cambridge Institute of Public Health, University Forvie Site, Robinson Way, Cambridge, CB2 0SR, United Kingdom

Please address correspondence to:

Dr Louise Marston

Department of Primary Care and Population Health

For peer review only

University College London

Rowland Hill Street

London

NW3 2PF

United Kingdom

l.marston@ucl.ac.uk

Telephone: +44 20 7794 0500 (36768)

Fax: +44 20 7794 1224

Words: 3,~~166~~376

For peer review only

ABSTRACT

Objectives: To investigate how smoking status is recorded in UK primary care; to evaluate ~~if~~ whether appropriate multiple imputation (MI) of smoking status yields results consistent with health surveys.

Setting: UK primary care and a population survey conducted in the community.

Participants: We identified 354,204 patients aged 16 or over in The Health Improvement Network (THIN) primary care database registered with their general practice 2008-2009 and 15,102 individuals aged 16 or over in the Health Survey for England (HSE).

Outcome measures: Age-standardised and~~,~~ age-specific proportions of smokers, ex-smokers and non-smokers in THIN and the HSE before and after multiple imputation (MI). Using information on time since quitting in the HSE, we ~~extrapolated~~ estimated when ex-smokers ~~may be considered~~are typically recorded as non-smokers in primary care records.

Results: In THIN, smoking status was recorded for 84% of patients within one year of registration. Of these; 28% were smokers (21% in the HSE). After MI of missing smoking data, the proportion of smokers was 25% (missing at random) and 20% (missing not at random). With increasing age, more were identified as ex-smokers in the HSE than THIN. It appears that those who quit ~~before the ages~~

For peer review only - http://bmjopen.bmj.com/site/about/guidelines.xhtml

~~of 25-30 years~~before age 30 were less likely to be recorded as an ex-smoker in primary care than people who quit later.

Conclusions: Smoking status ~~is~~ was relatively well recorded in primary care. Misclassification of ex-smokers as non-smokers is likely to occur in those quitting smoking at an early age and/ or a long time ago. Those with no smoking status information are more likely to be ex or non-smokers ~~versus~~ than smokers.

STRENGTHS AND LIMITATIONS OF THIS STUDY

- This study includes data from ‘real’ life primary care electronic records
- First study to compare the definition of smoking status in primary care ~~versus~~ with a population survey
- Study focuses on data recorded in the first year after patient registration and may not be applicable to other times.

KEYWORDS: recording of smoking, primary care databases, Health Survey for England, missing data, multiple imputation

For peer review only - http://bmjopen.bmj.com/site/about/guidelines.xhtml

INTRODUCTION

A fifth of the British adult population are smokers [1] and there is still a need for further research into smoking and smoking related diseases including coronary heart disease and stroke, respiratory diseases and cancers. Routinely collected smoking data can be used in clinical practice to identify populations at risk of smoking-related diseases, such as identifying smokers to have undergo spirometry testing to identify detect those withfor early diagnosis of Chronic Obstructive Pulmonary Disease (COPD), or to be invited for smoking cessation services. It is important to understand the accuracy of the data, and whether cases may be missed in those with no recorded smoking status. Electronic health records, including primary care databases, have proved to be very powerful resources for epidemiological and health research.[2-12], Electronic health records also allowallowing research that would be difficult to capture using primary research methods; for example, studying the elderly and people with severe mental illness.[4, 7, 9, 11] Additionally they include millions of patients giving power to study rare conditions. Nevertheless, as they are collected for clinical reasons, they raise a number of issues when used for research; not least of these is missing data.

In order to conduct such research, it is important to understand how smoking status is recorded in primary care and how missing data may be addressed.. There is evidence that the recording of smoking status has improved substantially in UK primary care[13, 14] and estimates of *current smoking* are

Formatted: Font: Italic

For peer review only - http://bmjopen.bmj.com/site/about/guidelines.xhtml

similar to large population surveys.[15, 16] and mMost general practices now routinely record smoking status at regular intervals as a part of the Quality Outcome Framework.[1517] However, we do not know how the different and non-standardised classifications of *ex and, non and current smokers* in primary care records compared to the standardised recording of smoking status in population surveys such as the Health Survey for England (HSE).

Formatted: Font: Italic

In additionAs noted already, a proportion of patients still lack a smoking status record in their primary care records. It is unclear how to deal with these patients when conducting research where smoking status is either the outcome of the research or an explanatory factor for patients' health.[3, 6, 1618, 1719] Methodological research has demonstrated that including only patients with complete records can substantially bias the results, especially when the reason for missing data is associated with patient outcomes.[1820, 21] In recent years, efforts have been made to address missing data in primary care databases[3, 17, 19, 22] using multiple imputation, though reporting on the comparability of the results of multiple imputation with population data has been sparse. Therefore, it is unclear whether multiple imputation accurately replicates data representing the population.[3, 6, 1719, 2023] Our previous work on missing data in The Health Improvement Network (THIN) primary care database showed that many health indicator measurements (for example, weight and blood pressure) recorded within the first year of patients' registration at a general practice were comparable with large external datasets before and after multiple imputation.[1618] However,

For peer review only - http://bmjopen.bmj.com/site/about/guidelines.xhtml

smoking status was not directly comparable with data from the Health Survey for England (HSE). Although the proportion of smokers was similar between THIN and the HSE *before* multiple imputation of data in THIN, the proportion of smokers was substantially higher *after* multiple imputation in THIN. On the other hand, the proportion of ex-smokers was substantially lower in THIN both *before* and *after* imputation compared to the HSE. This suggests that current smokers may be adequately identified using primary care data and most people with missing data on smoking status are likely to be either ex or non-smokers. This has clinical importance as smoking status (including ex-smoking) may be used to identify those at risk of disease, for example chronic obstructive pulmonary disease or cardiovascular disease.

In this study we further investigate recording of smoking status in primary care and explore potential reasons for the discrepancy in the proportion of ex-smokers between primary care records and the HSE. Specifically, we seek to deduce when ex-smokers may not be recorded as such in primary care records based on information about time since quitting in the HSE. Finally, we aim to provide a practical solution for imputation of missing smoking status records in routinely collected clinical data.

METHODS

Study populations

For peer review only

We used data from THIN primary care database, from practices in England that had passed data quality checks, to ensure they were using their computer system to record all patient consultations.[~~21~~24-26] In the United Kingdom (UK) 98% of the population are registered with a National Health Service (NHS) general practitioner to receive routine healthcare.[~~22~~27] THIN is broadly representative of all general practices in the UK in terms of age and sex of patients, practice size and geographical distribution.[~~23~~28] The database contains information on socio demographics, symptoms, diagnoses, referrals to secondary care, prescribing, results of tests and health status indicators. The data provider (CSD-MR) obtained overall ethical approval from the South East MREC (MREC/03/01/073) and this study was further approved by a THIN scientific review committee.

For this study we selected patients aged 16 years or over who registered with a general practice between 1st January 2008 and 31st December 2009 (N=354,204) and were registered for at least a year. ~~w~~We examined records from the first year after the patient registered, hence using data up to the end of 2010. Many people have a “new patient check” soon after registration, where information on demographics, health indicators and disease status is collected.

We compared the distribution of smoking status with that in the HSE from 2008 for those aged 16 years or over (N=15,102). The HSE is a national annual cross sectional interview based survey of approximately 22,000 people.[~~24~~29] The

For peer review only

For peer review only - http://bmjopen.bmj.com/site/about/guidelines.xhtml

survey includes questions on socio demographics, general health and information on smoking status. The HSE has nearly complete records of smoking (99.3%) and we therefore used the data from patients with complete smoking information.

Definition of smoking status

In THIN, smoking status was recorded by self-report. In many general practices this would be on the basis of a questionnaire submitted at the time of registration, whereas in other general practices this would be recorded in conjunction with a clinical consultation with the general practitioner or practice nurse. GPs and nurses may be more interested in the separation between current non-smokers and smokers, thus the non-smoking categories may include some people who are never smokers as well as some who are ex-smokers in primary care records. Patients would be classed as current non-smoker, or current smokers. In some instance the non-smokers would be classified as ex-smokers but this was variably defined from one practice to another. In THIN we extracted smoking status data either using Read codes[30] which were classified into non-smoker, ex-smoker and smoker with clinical input, or we used the categorisation (non-smoker, ex-smoker or current smoker) provided in the Additional Health Data. In the HSE, smoking status was defined on the basis of a series of questions (see Appendix 1) and individuals who had ever smoked (but did not smoke at the time of the interview) would be defined as ex-smokers, regardless of their age at quitting and length of time since they quit. The HSE holds information on when

For peer review only - http://bmjopen.bmj.com/site/about/guidelines.xhtml

ex-smokers quit so that age at the time they quit can be deduced, whereas this information was not consistently available in THIN.

Statistical analyses

Initially, we examined smoking status (smoker, ex-smoker, non-smoker or missing) in THIN and the HSE, overall, by age group, gender and Index of Multiple Deprivation 2004 (IMD) quintile[2531]. Then we used multiple imputation to impute missing smoking status data in THIN. Multiple imputation with chained equationsvia full conditional specification was performed using Stata's "ice" command. [32, 33] Multiple imputation is a statistical method which uses the data available to model the likely distribution of missing data.[18]20] A number of imputed datasets are produced in each of which plausible values are drawn from the imputation model. The method is designed to correctly reflect the uncertainty surrounding the missing values. With an appropriate imputation model, multiple imputation is an unbiased method of accounting for missing data. It is usually performed under the missing at random (MAR) assumption, but it may also be performed under specific missing not at random (MNAR) assumptions. These methods have been described in greater detail elsewhere.[1820, 26-2834-36]

After preliminary analysis,[2634] we included the following variables in the multiple imputation models: age in years, gender and IMD quintile,[2531] health indicators: smoking status (three categories, non, ex and current smoker), height,

For peer review only - http://bmjopen.bmj.com/site/about/guidelines.xhtml

weight, systolic and diastolic blood pressures and disease indicators: type II diabetes, coronary heart disease (CHD) and cerebrovascular accident (CVA). There were missing values for smoking status, blood pressure, weight, height and IMD quintile. Multiple imputation was performed using Chained Equations using the ice command using Stata 11.[29, 30] Within the full conditional specification imputation algorithm, cContinuous variables were imputed using multiple linear regression, smoking status using multinomial regression and IMD quintile using ordered logistic regression. Percentages in each smoking category were obtained using Rubin's Rules.[3137] In the first multiple imputation we assumed that smoking data were MAR and hence allowed imputed smoking data of either smokers, non-smokers or ex-smokers (using a MAR assumption; hereafter referred to as MAR MI). In the second multiple imputation we assumed that all smokers had been recorded (so that smoking data were MNAR) and we imputed missing smoking data as either ex-smokers or non-smokers (hereafter referred to as MNAR MI).

Following multiple imputation we carried out age-specific direct standardisation using the HSE as the standard population and the age-specific proportion in each smoking category from THIN. This was done to account for the fact that the mean age in the HSE was 49 years while the mean age in THIN was 38 years in the year after registration.

For peer review only - http://bmjopen.bmj.com/site/about/guidelines.xhtml

We deduced the average time after which an ex-smoker is no longer classified as an ex-smoker in primary care records by combining information from the HSE on when ex-smokers quit and the age-specific distribution of ex-smokers in THIN after imputation of non and ex-smokers. This was done by ranking the individuals in the HSE in accordance to the length of time since they quit by 10 year age groups and then 'reclassifying' individuals who had quit the longest time ago within each age group from ex to non until we reached the same proportion of ex-smokers in the HSE as in THIN. By doing this, we were able to estimate the average time that elapses from quitting smoking after which true ex-smokers are recorded as non-smokers in primary care records.

RESULTS

In total, 354,204 individuals were included from 366 general practices in THIN and 15,102 individuals from the HSE. Individuals in THIN were, on average 11 years younger than those in the HSE (38 years versus 49 years, respectively) (Table 1). Smoking status was recorded for 84% in THIN within one year of initial registration. Before multiple imputation of missing data, a greater proportion of people were recorded as smokers in THIN than the HSE (24% versus 21% respectively), and the proportions of ex-smokers and non-smokers differed substantially between THIN and the HSE (Table 1).

For peer review only - http://bmjopen.bmj.com/site/about/guidelines.xhtml

Table 1: Summary statistics for THIN in the first year of registration and the HSE 2008

Variable	THIN n	THIN %	HSE n	HSE %
Male	164,085	46	6,760	45
Female	190,119	54	8,342	55
Missing sex		0		0
Non-smoker	165,618	47	7,874	52
Ex-smoker	49,874	14	3,966	26
Current smoker	83,526	24	3,158	21
Missing smoking status	55,186	16	104	1
Age years mean (SD)	38	(17)	49	(19)
Missing age		0		0
Least deprived	69,104	20	3,321	22
Quintile 2	71,771	20	3,039	20
Quintile 3	66,422	19	3,010	20
Quintile 4	71,789	20	2,928	19
Most deprived	52,120	15	2,804	19
Missing IMD	22,998	6	0	0

Abbreviations: HSE Health Survey for England 2008; THIN The Health Improvement Network.

Our first analyses used missing as a separate category of smoking, so we refer to those with reported smoking status as “known smokers” and “known ex-smokers”. The proportion of known smokers by age group was similar in THIN and the HSE between 30 and 79 years, but this was not the case for the proportions of known ex-smokers and non-smokers (Figure 1). In the HSE, the proportion of ex-smokers increased from 12% within the 20-29 age group to 46% in the 80-89 age group. In THIN, the proportion of known ex-smokers also increased with age although the overall proportion of known ex-smokers was smaller than in the HSE for all age groups after 20-29 years. Conversely, in the HSE, the proportion of non-smokers decreased slightly from 56% in the 20-29

For peer review only - http://bmjopen.bmj.com/site/about/guidelines.xhtml

age group to 48% in the 80-89 age group. Within THIN, the proportion of known non-smokers remained constant with increasing age at around 43%. The proportion of missing smoking data in THIN was relatively constant at less than 20% until the 70-79 years age group, but increased substantially thereafter (Figure 1).

(Figure 1 here)

In THIN, the percentage of non-smokers was greater for women (52%) than men (40%) while the percentage of known smokers was smaller for women (21%) than men (27%). There were similar trends in the HSE, although the percentage differences between sexes were smaller (smokers: 22% of men versus 20% of women).

The proportions in each smoking status category varied substantially by social deprivation in both THIN and the HSE (Figure 2). In THIN, the percentage of non-smokers decreased from 52% in the least deprived quintile to 40% in the most deprived quintile. The percentage of known ex-smokers decreased slightly with increasing deprivation. In contrast, the percentage of known smokers increased with increasing deprivation from 16% in the least deprived quintile to 34% in the most deprived quintile (Figure 2). The patterns were similar in the HSE although the proportion of ex-smokers was substantially larger across all levels of deprivation in the HSE compared to THIN.

For peer review only - http://bmjopen.bmj.com/site/about/guidelines.xhtml

For peer review only

(Figure 2 here)

Analyses imputing missing smoking status

After MAR MI of THIN, age-standardised smoking prevalences still differed somewhat between THIN and the HSE. For example, 22% were ex-smokers in THIN compared with 26% in the HSE; 25% were smokers in THIN, compared with 21% in the HSE (Table 2).

After MNAR MI of THIN (that is, regarding specifying that missing values as are either ex-smokers or non-smokers), the age-standardised prevalence of smoking in THIN was similar to that in the HSE (Table 2). However, the age-specific prevalence of ex-smokers was still greater in the HSE than in THIN. Age-specific analysis showed that this difference was greatest at older ages, and indeed reversed at younger ages. This suggested that individuals who had quit in the less recent past might be classified as non-smokers in THIN but as ex-smokers in HSE.(Figure 3).

For peer review only - http://bmjopen.bmj.com/site/about/guidelines.xhtml

Table 2: Percentages within each smoking status for THIN and the HSE 2008 after various adjustments

Category	THIN			HSE	
	Complete ~~case~~records	After MAR MI.[ab]	After MNAR MI[ac]	Observed	Reclassifying ex-smokers[d]
	%	%	%	%	%
Non-smoker	55	53	57	53	57
Ex-smoker	17	22	23	26	22
Smoker	28	25	20	21	21

Abbreviations: HSE Health Survey for England 2008; THIN The Health Improvement Network.
[a] Directly standardised using the HSE age distribution as standard.
[b] Imputed assuming that missing values are smokers, non-smokers or ex-smokers
[c] Imputed assuming that missing values are non-smokers or ex-smokers
[d] Within each age group, reclassifying the optimum number of ex-smokers as non-smokers based on the distributions shown after MNAR MI.

(Figure 3 here)

The median time since ex-smokers quit in the HSE varied greatly by age group (Table 3), from two years (Interquartile range (IQR): 0, 3) in the under 20s to 40 (IQR: 25, 51) years in those aged 90 or over (Table 3). Equating proportions of ex-smokers in THIN to that in the HSE data suggested the typical time-window after which patients are no longer regarded as ex-smokers in primary care, but instead regarded as non-smokers, varied with age. Thus, typically individuals who registered with a general practice when they were in their forties would no longer be recorded as an ex-smoker if they quit more than 22 years earlier (when they were between 18 and 27 years of age) (Table 3). Individuals registering in their seventies would typically no longer be recorded as ex-smokers if they quit 42 years earlier (when they were between the ages of 28 and 37 years) (Table 3). Yet, most individuals who quit after the age of 30 would still be captured as

For peer review only - http://bmjopen.bmj.com/site/about/guidelines.xhtml

ex-smokers when they later registered with a new general practice. Using these age-specific extrapolations to reclassify ex-smokers as non-smokers in the HSE according to when they quit, we can see that the age-specific distributions of ex-smokers in THIN and the reclassified HSE are similar (Figure 3).

Table 3: Age specific centiles of time since quitting smoking in the HSE 2008

Age group	Median time since quitting (years)	Extrapolated number of years since quitting	Extrapolated age when they quit
<20	2	*	*
20-29	3	*	*
30-39	5	14	16 - 25
40-49	10	22	18 - 27
50-59	20	30	20 - 29
60-69	24	35	25 - 34
70-79	30	42	28 - 37
80-89	32	40	40 - 49
90+	40	46	44+

*Not possible to assign an optimal value for reclassification to these age groups
Abbreviations: HSE Health Survey for England 2008

DISCUSSION

The proportion of newly registered patients in THIN between 2008 and 2009 with a record of being a smoker was slightly higher than the HSE in 2008. However, the proportion of individuals recorded as ex-smokers and non-smokers differed substantially between THIN and the HSE. Overall, a larger proportion of individuals were recorded as ex-smokers in the HSE than in THIN and this increased with age. Likewise, the proportion of ex-smokers was substantially larger across all levels of deprivation in the HSE compared to THIN.

For peer review only

Under MAR MI there was a greater percentage of smokers (25%) and a smaller percentage of ex-smokers (22%) in THIN compared with the HSE (smokers 21%, ex-smokers 26%). However, ~~under~~ MNAR MI (assuming all missing data were either ex-smokers or non-smokers) slightly increased the proportion of non-smokers (57%) in THIN compared to the HSE (53%), whereas the proportion of ex-smokers (23%) was slightly lower in THIN. Moreover, the latter imputation resulted in a relatively larger percentage of ex-smokers in THIN in those aged under 30 years compared with the HSE. This may be because the imputation model was unable to distinguish between ex and non-smokers in those age groups as both are unlikely to have developed typical later onset diseases which are key predictors of smoking status in the imputation model.

There may be several reasons for the discrepancy in the distribution of the smoking categories between THIN and the HSE. In the HSE, the definition of an ex-smoker was highly sensitive and clearly defined.[~~24~~29] Thus respondents were categorised as ex-smokers even if they were a trivial smoker, smoked for a short period of time and/ or quit many decades ago. Also, the HSE used computer aided personal interviewing; where questions were read to the respondent in a standardised way from the screen and a detailed sequence of questions were asked to ascertain current smoking status. In primary care, while smoking status is systematically recorded in medical records, there is no detailed protocol for recording smoking status and the ascertainment is thus likely to vary by how the information was obtained. Many practices use self-report

For peer review only - http://bmjopen.bmj.com/site/about/guidelines.xhtml

questionnaires at registration including smoking status. Smoking status is then updated by health professionals (general practitioners and/ or practice nurses) during consultations where smoking status is often recorded as part of an assessment of current or future disease risk.

Our examination of the age-standardised data suggests that typically an ex-smoker in primary care settings is recorded as a non-smoker when they quit at a young age or had not smoked for a substantial time period. This could be because the patient may not volunteer previous smoking in either initial self-report questionnaire or on questioning by clinicians when it was minor, long ago or they consider it not relevant to their current or future health. It is possible that patients are more reluctant to volunteer ex-smoking habits when data are being held on their medical record and is not anonymous. However, comparing the proportion of individuals with a smoking record in THIN with that of the HSE we found a similar distribution suggesting that most smokers were identified in the first year of their registration in primary care. Similar findings have been observed in the literature by calendar year.[3218] While some studies suggest underreporting of smoking among pregnant women in primary care[33] we found no evidence this was a general pattern. With the introduction of the Quality and Outcomes Framework in 2004, there has also been increased incentive to identify smokers in relation to specific disease outcomes.[3438, 3539] Indeed we found in our previous study that those with respiratory and cardiac conditions were more likely to have any smoking status recorded within the first year of

For peer review only - http://bmjopen.bmj.com/site/about/guidelines.xhtml

registration.[13] Smoking status was validated in the HSE in 2007 by the use of saliva cotinine samples and was found to be accurate[~~36~~40].

The method of age standardisation then deducing the average time since quitting and reclassifying them to non-smokers in the HSE is relatively crude and assumes that everyone who becomes an ex-smoker does so at the same time in their lives as others in their age group. However, it ~~may~~ is likely to be indicative of reporting of smoking status at the GP practice, given the results shown in this study.

An alternative method of dealing with unobserved smoking data is to dichotomise smoking status into current smokers and non-current smokers with missing data assumed to be non-current smokers. However, it should be noted that this solution may be to the detriment of some epidemiological studies where ex-smokers who quit recently are at greater risk of disease than non-smokers. For example, the 50 year follow up of male British doctors shows that ex-smokers had elevated age standardised mortality rates for many diseases.[~~37, 38~~41, 42]

Our findings suggest that *in contrast* to health surveys, patients who quit smoking at a young age (before 25-30) are likely to be recorded by their general practice as a non-smoker instead of an ex-smoker. This has implications for researchers using these data sources. To our knowledge this is the first study which seeks to deduce and quantify typical time between when a smoker quit and when they are

For peer review only

For peer review only - http://bmjopen.bmj.com/site/about/guidelines.xhtml

no longer perceived as an ex-smoker in primary care. Clinicians, policy-makers and researchers who wish to use smoking status in primary care records to identify populations at risk of smoking-related diseases can be reassured by our findings that using data from new registrations, most current smokers will be identified and misclassification of ex-smokers is more likely to have occurred in those who have quit smoking at an early age and/ or a long time ago.

For peer review only

Figure Legends

Figure 1: Smoking status percentages in THIN and the HSE 2008 by age group

Figure 1 footnotes:
Solid line is the Health Survey for England 2008, dashed line is The Health Improvement Network (THIN)

Figure 2: Smoking status percentages in THIN and the HSE 2008 by deprivation quintile

Figure 2 footnotes:
*IMD 1 is the least deprived and IMD 5 is the most deprived
Darker bars represent the HSE 2008, lighter bars represent THIN
Abbreviations: HSE Health Survey for England 2008, IMD Index of multiple deprivation, THIN The Health Improvement Network

Figure 3: Age group specific percentages of ex-smokers in THIN (after MNAR imputation) and the HSE 2008 (before and after reclassifying ex-smokers in the HSE who quit before the age specified in Table 3 column 3 to be non-smokers)

Figure 3 footnotes:
Abbreviations: THIN The Health Improvement Network, HSE Health Survey for England 2008

For peer review only

For peer review only - http://bmjopen.bmj.com/site/about/guidelines.xhtml

Conflict of interest and funding

The authors have no conflicts of interest to declare. This study is funded by a UK Medical Research Council grant [G0900701]. The funder had no influence over the study design, results or decision to publish this work. JRC was funded by a UK Economic and Social Research Council research fellowship grant [RES-063-27-0257]. IRW was funded by a United Kingdom Medical Research Council grant [U105260558].

Author contributions

LM extracted and analysed the data and wrote the first draft of the paper with help from IP and JRC. KRW and IN provided clinical input and IRW and RWM provided additional statistical input. All authors commented on the paper and helped write subsequent drafts.

Data sharing statement

No data are available

I Dr Louise Marston the Corresponding Author of this article contained within the original manuscript which includes any diagrams & photographs, other illustrative material, video, film or any other material howsoever submitted by the Contributor(s) at any time and related to the Contribution ("the Contribution") have the right to grant on behalf of all authors and do grant on behalf of all authors, a licence to the BMJ Publishing Group Ltd and its licensees, to permit this Contribution (if accepted) to be published in BMJ Open and any other BMJ Group products and to exploit all subsidiary rights, as set out in the licence at: (http://group.bmj.com/products/journals/instructions-for-authors/BMJOpen_licence.pdf)

For peer review only - http://bmjopen.bmj.com/site/about/guidelines.xhtml

References

1 Office for National Statistics Opinions and Lifestyle Survey, Smoking Habits Amongst Adults, 2012; 2013 (http://www.ons.gov.uk/ons/dcp171776_328041.pdf) (accessed January 2014)

2 Davies AR, Smeeth L, Grundy EMD. Contribution of changes in incidence and mortality to trends in the prevalence of coronary heart disease in the UK: 1996-2005. *Eur Heart J* 2007;2142-2147.

3 Delaney JAC, Daskalopoulou SS, Brophy JM et al Lifestyle variables and the risk of myocardial infarction in the general practice research database (electronic article). *BMC Cardiovasc Disord* 2007;38.

4 Douglas IJ, Smeeth L. Exposure to antipsychotics and risk of stroke: self controlled case series study (electronic article). *Br Med J* 2008;

5 Gelfand JM, Neimann AL, Shin DB et al Risk of myocardial infarction in patients with psoriasis. *JAMA* 2006;1735-1741.

6 Hippisley-Cox J, Coupland C, Vinogradova Y et al Derivation and validation of QRISK, a new cardiovascular disease risk score for the United Kingdom: prospective open cohort study. *Br Med J* 2007;136-141.

For peer review only - http://bmjopen.bmj.com/site/about/guidelines.xhtml

7 Osborn DPJ, Levy G, Nazareth I et al Relative risk of cardiovascular and cancer mortality in people with severe mental illness from the United Kingdom's General Practice Research Database. *Arch Gen Psychiatry* 2007;242-249.

8 Smeeth L, Thomas SL, Hall AJ et al Risk of myocardial infarction and stroke after acute infection or vaccination. *N Engl J Med* 2004;2611-2618.

9 Walters K, Rait G, Petersen I et al Panic disorder and risk of new onset coronary heart disease, acute myocardial infarction, and cardiac mortality: cohort study using the general practice research database. *Eur Heart J* 2008;2981-2988.

10 Horsfall LJ, Rait G, Walters K et al Serum Bilirubin and Risk of Respiratory Disease and Death *JAMA* 2011;691-697

11 Kiri VA, Fabbri LM, Davis KJ et al Inhaled corticosteroids and risk of lung cancer among COPD patients who quit smoking *Respir Med* 2009;85-90

12 Horsfall LJ, Nazareth I, Petersen I. Cardiovascular Events as a Function of Serum Bilirubin Levels in a Large Statin-Treated Cohort. *Circulation* 2012;2556-2564

For peer review only - http://bmjopen.bmj.com/site/about/guidelines.xhtml

13 Szatkowski L, Lewis S, McNeill A et al Is smoking status routinely recorded when patients register with a new GP? *Fam Pract* 2010;673–675

14 Dhalwani NN, Tata LJ, Coleman T, Fleming KM, Szatkowski L Completeness of Maternal Smoking Status Recording during Pregnancy in United Kingdom Primary Care Data. *PLoS One* 2013;e72218

15 Szatkowski L, Lewis S, McNeill A et al Can data from primary care medical records be used to monitor national smoking prevalence? *J Epidemiol Community Health* 2012;791-795.

16 Langley TE, Szatkowski LC, Wythe S, et al Can primary care data be used to monitor regional smoking prevalence? An analysis of The Health Improvement Network primary care data *BMC Public Health* 2011, 11:773

~~15~~ 17 Coleman T, Lewis S, Hubbard R et al Impact of contractual financial incentives on the ascertainment and management of smoking in primary care. *Addiction* 2007;102:803e8.

~~16~~ 18 Marston L, Carpenter JR, Walters KR et al Issues in multiple imputation of missing data for large general practice clinical databases *Pharmacoepidemiol Drug Saf* 2010;618–626

For peer review only - http://bmjopen.bmj.com/site/about/guidelines.xhtml

~~17~~ 19 Weiner MG, Barnhart K, Xie D et al Hormone therapy and coronary heart disease in young women. *Menopause* 2008;86-93.

~~18~~ 20 Sterne JAC, White IR, Carlin JB et al. Multiple imputation for missing data in epidemiological and clinical research: potential and pitfalls. *Br Med J* 2009;b2393

21 Carpenter J, Kenward M Multiple imputation and its application 2013 Wiley

~~19~~ 22 Hippisley-Cox J, Coupland C, Vinogradova Y et al Predicting cardiovascular risk in England and Wales: prospective derivation and validation of QRISK2. *Br Med J* 2008;1475-1482

~~20~~ 23 Collins GS, Altman DG An independent and external validation of QRISK2 cardiovascular disease risk score: a prospective open cohort study *Br Med J* 2010;c2442

~~21~~ 24 The Health Improvement Network The Health Improvement Network. London: The Health Improvement Network; 2014 (http://csdmruk.cegedim.com/) (Accessed January 2014).

Formatted: Font: (Default) Arial, 12 pt, Not Bold

For peer review only - http://bmjopen.bmj.com/site/about/guidelines.xhtml

25 Horsfall L, Walters K, Petersen I.Identifying periods of acceptable computer usage in primary care research databases. *Pharmacoepidemiol Drug Saf* 2013; 64-69

26 Maguire A, Blak BT, Thompson M. The importance of defining periods of complete mortality reporting for research using automated data from primary care. *Pharmacoepidem Drug Safe* 2009; 76-83.

~~22~~ 27 Lis Y, Mann RD The VAMP Research multi-purpose database in the U.K. *J Clin Epidemiol* 1995;431-443.

~~23~~ 28 Blak BT, Thompson M, Dattani H, ~~Bourke A~~et al Generalisability of The Health Improvement Network (THIN) database: demographics, chronic disease prevalence and mortality rates. *Inform Prim Care* 2011;251-255.

~~24~~ 29 National Centre for Social Research and University College London. Department of Epidemiology and Public Health, *Health Survey for England, 2008* [computer file]. *2nd Edition.* Colchester, Essex: UK Data Archive [distributor], October 2010. SN: 6397

30 Booth N What are the Read Codes? *Health Libr Rev* 1994;177-82

For peer review only - http://bmjopen.bmj.com/site/about/guidelines.xhtml

~~25~~ 31 Noble M, Wright G, Dibben C et al Indices of Deprivation 2004. Report to the Office of the Deputy Prime Minister. London: Neighbourhood Renewal Unit; 2004 (http://webarchive.nationalarchives.gov.uk/20120919132719/http://www.communities.gov.uk/documents/communities/pdf/131209.pdf) (Accessed January 2014)

32 Stata Corporation. *Stata Statistical Software: Release 11*. College Station, TX: Stata Corporation; 2009

33 Royston P Multiple imputation of missing values: Update of ice. *Stata Journal* 2005;527-536.

~~26~~ 34 Spratt M, Carpenter J, Sterne JAC et al Strategies for Multiple Imputation in Longitudinal Studies *Am. J. Epidemiol.* 2010;478-487

~~27~~ 35 White IR, Royston P, Wood A Multiple imputation using chained equations: issues and guidance for practice (tutorial). *Stat Med* 2011;377-399

~~28~~ 36 Graham JW Missing data analysis: Making it work in the real world *Annu Rev Psychol* 2009;549-576

~~29 Stata Corporation. *Stata Statistical Software: Release 11*. College Station, TX: Stata Corporation; 2009~~

For peer review only - http://bmjopen.bmj.com/site/about/guidelines.xhtml

~~30 Royston P Multiple imputation of missing values: Update of ice. *Stata Journal* 2005;527-536.~~

~~31~~ 37 Rubin DB *Multiple imputation for non-response in surveys*. New York: John Wiley and Sons; 1987

~~32 Szatkowski L, Lewis S, McNeill A et al Can data from primary care medical records be used to monitor national smoking prevalence? *J Epidemiol Community Health* 2011. doi:10.1136/jech.2010.120154~~

~~33 Shipton D, Tappin DM, Vadiveloo T et al Reliability of self reported smoking status by pregnant women for estimating smoking prevalence: a retrospective, cross sectional study *Br Med J* 2009;b4347.~~

~~34~~ 38 The British Medical Association and NHS Employers Quality and Outcomes Framework guidance for GMS contract 2011/12; 2011 (http://www.nhsemployers.org/Aboutus/Publications/Documents/QOF_guidance_GMS_contract_2011_12.pdf) (Accessed January 2014)

~~35~~ 39 Campbell S, Reeves D, Kontopantelis E et al Quality of Primary Care in England with the Introduction of Pay for Performance *N Engl J Med* 2007;181-190

For peer review only

For peer review only - http://bmjopen.bmj.com/site/about/guidelines.xhtml

~~36~~ 40 Wardle H, Mindell J Adult cigarette smoking. In Craig R, Shelton N (Ed.), Health Survey for England 2007. Volume 1. Healthy lifestyles: Knowledge, attitudes and behaviour. 2008;149-176 NHS Information Centre.

~~37~~ 41 Doll R, Peto R, Boreham J et al Mortality in relation to smoking: 50 years' observations on male British doctors *Br Med J* 2004, doi:10.1136/bmj.38142.554479.AE

~~38~~ 42 Kenfield SA, Wei EK, Rosner BA et al Burden of smoking on cause-specific mortality: application to the Nurses' Health Study *Tob Control* 2010;248-254

For peer review only

For peer review only - http://bmjopen.bmj.com/site/about/guidelines.xhtml

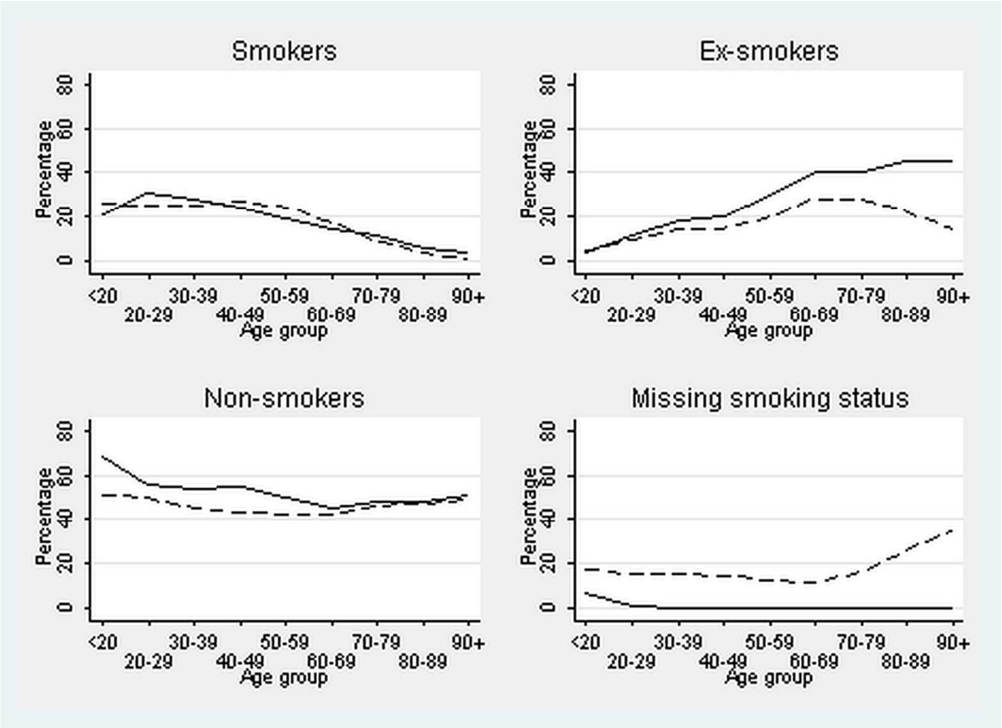

Figure 1: Smoking status percentages in THIN and the HSE 2008 by age group

Solid line is the Health Survey for England 2008, dashed line is The Health Improvement Network (THIN)

90x65mm (300 x 300 DPI)

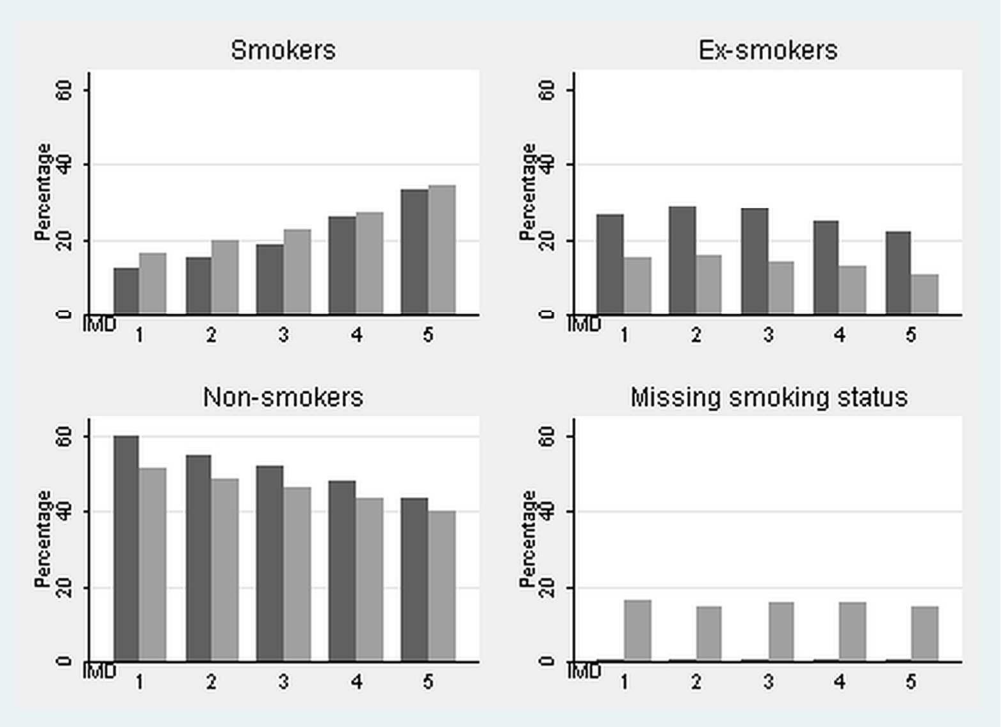

Figure 2: Smoking status percentages in THIN and the HSE 2008 by deprivation quintile

*IMD 1 is the least deprived and IMD 5 is the most deprived
Darker bars represent the HSE 2008, lighter bars represent THIN
Abbreviations: HSE Health Survey for England 2008, IMD Index of multiple deprivation, THIN The Health Improvement Network

90x65mm (300 x 300 DPI)

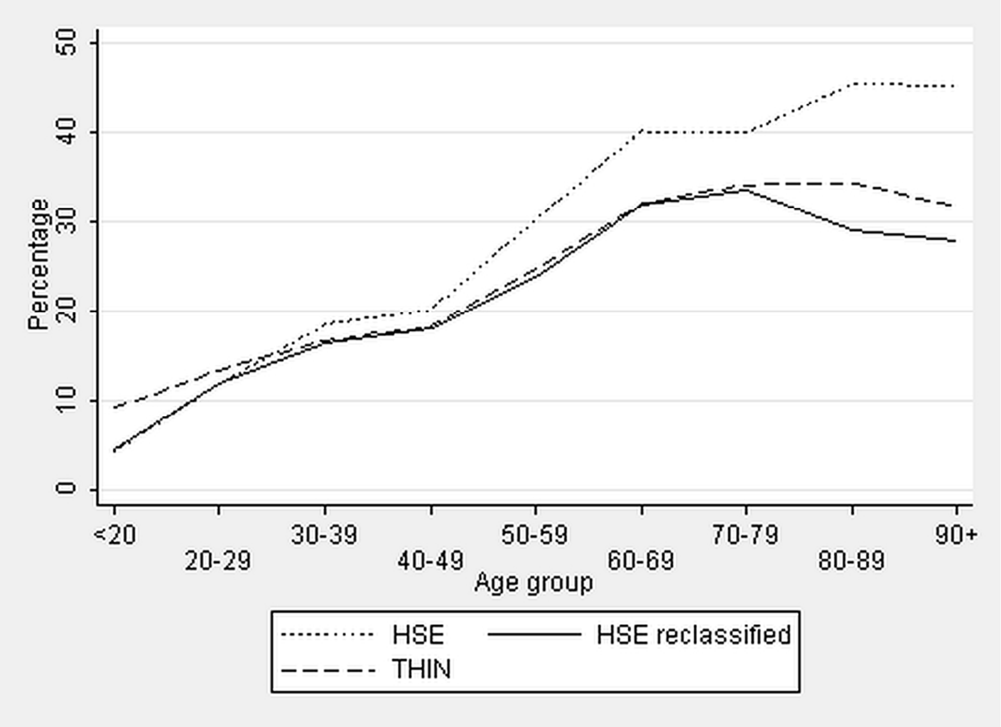

Figure 3: Age group specific percentages of ex-smokers in THIN (after MNAR imputation) and the HSE 2008 (before and after reclassifying ex-smokers in the HSE who quit before the age specified in Table 3 column 3 to be non-smokers)

Abbreviations: THIN The Health Improvement Network, HSE Health Survey for England 2008

90x65mm (300 x 300 DPI)

Appendix 1

For the Health Survey for England 2008 analysis, we used the derived variable cigsta3. This is a three category variable non-smokers, ex-smokers, current smokers. It is derived from a number of questions asked in the Health Survey for England as follows:

If the participant

- Reported never smoking in the constituent questions ("May I just check, have you ever smoked a cigarette, a cigar or a pipe?", "Have you ever smoked cigarettes?" (the latter only asked to those who have smoked but does not smoke cigarettes nowadays)), they were coded as a never regular smoker on this variable.
- Using: "Did you smoke cigarettes regularly, that is at least one cigarette a day, or did you smoke them only occasionally?", if participants answered "regularly", they are coded as an ex-smoker. If they responded "occasionally" or "only tried once or twice", they were categorised as a never regular smoker
- If participants answered "yes" to "Do you smoke cigarettes at all nowadays?" this was taken as "current smoker" in the variable cigsta3.
- If participants gave no answer/ refused to any of the constituent questions, this was carried forward to cigsta3
- If participants responded don't know to any of the constituent questions, this was carried forward to cigsta3
- If participants answered not applicable to "Do you smoke cigarettes at all nowadays?" this was carried forward to cigsta3.

National Centre for Social Research and University College London. Department of Epidemiology and Public Health, *Health Survey for England, 2008* [computer file]. *2nd Edition.* Colchester, Essex: UK Data Archive [distributor], October 2010. SN: 6397

For peer review only

STROBE 2007 (v4) Statement—Checklist of items that should be included in reports of *cross-sectional studies*

Section/Topic	Item #	Recommendation	Reported on page #
**Title and abstract**	1	(*a*) Indicate the study's design with a commonly used term in the title or the abstract	1
		(*b*) Provide in the abstract an informative and balanced summary of what was done and what was found	3-4
**Introduction**			
Background/rationale	2	Explain the scientific background and rationale for the investigation being reported	5-7
Objectives	3	State specific objectives, including any prespecified hypotheses	7
**Methods**			
Study design	4	Present key elements of study design early in the paper	7-8
Setting	5	Describe the setting, locations, and relevant dates, including periods of recruitment, exposure, follow-up, and data collection	7-8
Participants	6	(*a*) Give the eligibility criteria, and the sources and methods of selection of participants	8
Variables	7	Clearly define all outcomes, exposures, predictors, potential confounders, and effect modifiers. Give diagnostic criteria, if applicable	8-10
Data sources/ measurement	8*	For each variable of interest, give sources of data and details of methods of assessment (measurement). Describe comparability of assessment methods if there is more than one group	8-10
Bias	9	Describe any efforts to address potential sources of bias	*8*
Study size	10	Explain how the study size was arrived at	NA
Quantitative variables	11	Explain how quantitative variables were handled in the analyses. If applicable, describe which groupings were chosen and why	9-10
Statistical methods	12	(*a*) Describe all statistical methods, including those used to control for confounding	9-11
		(*b*) Describe any methods used to examine subgroups and interactions	9-11
		(*c*) Explain how missing data were addressed	9-11
		(*d*) If applicable, describe analytical methods taking account of sampling strategy	NA
		(*e*) Describe any sensitivity analyses	NA
**Results**			

For peer review only

Participants	13*	(a) Report numbers of individuals at each stage of study—eg numbers potentially eligible, examined for eligibility, confirmed eligible, included in the study, completing follow-up, and analysed	11
		(b) Give reasons for non-participation at each stage	NA
		(c) Consider use of a flow diagram	NA
Descriptive data	14*	(a) Give characteristics of study participants (eg demographic, clinical, social) and information on exposures and potential confounders	11-12
		(b) Indicate number of participants with missing data for each variable of interest	12
Outcome data	15*	Report numbers of outcome events or summary measures	12
Main results	16	(*a*) Give unadjusted estimates and, if applicable, confounder-adjusted estimates and their precision (eg, 95% confidence interval). Make clear which confounders were adjusted for and why they were included	12-16
		(*b*) Report category boundaries when continuous variables were categorized	NA
		(*c*) If relevant, consider translating estimates of relative risk into absolute risk for a meaningful time period	NA
Other analyses	17	Report other analyses done—eg analyses of subgroups and interactions, and sensitivity analyses	NA
**Discussion**			
Key results	18	Summarise key results with reference to study objectives	16
Limitations	19	Discuss limitations of the study, taking into account sources of potential bias or imprecision. Discuss both direction and magnitude of any potential bias	17-19
Interpretation	20	Give a cautious overall interpretation of results considering objectives, limitations, multiplicity of analyses, results from similar studies, and other relevant evidence	17-18
Generalisability	21	Discuss the generalisability (external validity) of the study results	19
**Other information**			
Funding	22	Give the source of funding and the role of the funders for the present study and, if applicable, for the original study on which the present article is based	21

*Give information separately for cases and controls in case-control studies and, if applicable, for exposed and unexposed groups in cohort and cross-sectional studies.

Note: An Explanation and Elaboration article discusses each checklist item and gives methodological background and published examples of transparent reporting. The STROBE checklist is best used in conjunction with this article (freely available on the Web sites of PLoS Medicine at http://www.plosmedicine.org/, Annals of Internal Medicine at http://www.annals.org/, and Epidemiology at http://www.epidem.com/). Information on the STROBE Initiative is available at www.strobe-statement.org.

For peer review only
